# LOTUS: Protocol for a double-blind placebo controlled randomized trial of hemp-derived cannabidiol for the treatment of cannabis use disorder

**DOI:** 10.1371/journal.pone.0308262

**Published:** 2024-09-30

**Authors:** L. Cinnamon Bidwell, Renee Martin-Willett, Samantha N. Melendez, Luiza Rosa, Gregory Giordano, Kent E. Hutchison, Angela D. Bryan

**Affiliations:** 1 Institute for Cognitive Science, University of Colorado Boulder, Boulder, CO, United States of America; 2 Psychology and Neuroscience, University of Colorado Boulder, Boulder, CO, United States of America; 3 Department of Psychiatry, University of Colorado Denver, Denver, CO, United States of America; PLoS ONE, UNITED STATES OF AMERICA

## Abstract

**Background:**

As cannabis legalization continues to spread across the United States, average Δ^9^-tetrahydrocannabinol concentrations in recreational products have significantly increased, and no prior study has evaluated effective treatments to reduce cannabis use among high potency cannabis users. Some research has found that the non-intoxicating cannabinoid cannabidiol reduces cannabis use and cannabis use disorder-related symptoms, such as affective disturbance and withdrawal. Results of these studies are promising but limited to synthetic or isolated forms of cannabidiol.

**Objective:**

Conduct a placebo-controlled randomized control trial comparing the effects of hemp-derived cannabidiol on reducing Δ^9^-tetrahydrocannabinol use in concentrate users with cannabis use disorder.

**Methods:**

**Design.** Double-blind, three-arm randomized placebo-controlled trial.

**Setting.** University in the Denver-Boulder, CO, USA area.

**Study population.** Community members who are heavy, stable cannabis concentrate users that meet criteria for at least moderate cannabis use disorder and are seeking to decrease or stop cannabis use.

**Data.** Self-report demographics, substance use, and mental health characteristics, blood and urine based biomarkers and anthropometrics.

**Outcomes.** Affective, physiological, and physical withdrawal symptoms, Δ^9^-tetrahydrocannabinol use.

**Analysis.** Three-group ANOVAs and χ^2^ tests will be used to compare baseline variables between groups. Characteristics that differ between groups will be evaluated as potential covariates in subsequent analyses. A multilevel modeling framework will be used for primary outcome analysis to account for the repeated observations nested within participants over time. Pairwise post-hoc simple effects tests will be conducted to confirm patterns of differences.

**Trial registration:**

ClinicalTrials.gov NCT06107062.

## Introduction

The *Cannabis sativa* L. plant contains hundreds of phytocannabinoids, but arguably of greatest importance to public health risk is the psychoactive cannabinoid Δ^9^-tetrahydrocannabinol (THC). THC is associated with risks for cannabis use disorder (CUD), cognitive harm, affective disturbance, and psychotomimetic symptoms [1]. In the wake of recreational cannabis legalization across the U.S., there has been an increase in the use of concentrated cannabis products (or “concentrates”) with THC concentrations as high as 90–95% [2, 3]. Concentrates popularity continues to rapidly increase; concentrates made up just 17% of the market in 2014 in Colorado [4], but had jumped to 35% only five years later.

Despite this explosive growth, very little research has been done to examine their effects. Current work suggests that high THC concentrations may increase the harms associated with cannabis use and contribute to greater dependence, withdrawal, and affective disturbance among concentrate users, over and above frequency of cannabis use [5–14]. Our and other groups’ data suggest that more frequent use of cannabis with higher THC concentration is associated with more severe CUD symptoms and is more likely to produce anxiety, agitation, paranoia, and psychosis [10]. Our prior work has also indicated that frequent concentrate users, relative to frequent flower users, report greater withdrawal, more total CUD symptoms, and are specifically more likely to endorse the Diagnostic and Statistical Manual of Mental Disorders, 5th edition (DSM-5) tolerance and loss of control diagnostic criteria [2]. Despite these risks, no previous work has explicitly evaluated effective treatments for reducing cannabis use and THC exposure in this group of high-risk users, who are most likely to benefit from an effective harm-reduction intervention.

One promising candidate CUD medication is the phytocannabinoid cannabidiol (CBD), which may regulate the reinforcing and motivational aspects of cannabis and other drugs [15]. CBD is a high-potency cannabinoid receptor-1 (CB_1_) and cannabinoid receptor-2 (CB_2_) inverse agonist that antagonizes the effects of full CB_1_ and CB_2_ agonists such as THC. Unlike THC, CBD has no intoxicating effects [16, 17], and little abuse liability among cannabis users [18]. CBD also increases availability of the endocannabinoid anandamide (AEA), potentially through inhibition of the AEA-hydrolyzing enzyme fatty acid amide hydrolase (FAAH) [19], and acts at a variety of other potentially relevant molecular targets, including the orphan G-protein-coupled receptor GPR55 and the 5-HT_1A_ receptor [20]. Given these mechanisms of action, CBD may have the potential to reduce THC withdrawal and reuptake.

For example, in a preclinical model of opioid dependence, a single 5 mg/kg dose of CBD inhibited heroin-seeking behavior during reinstatement [21]. Remarkably, this effect persisted for two weeks after CBD administration. When translated to opioid-dependent humans, a single oral CBD dose (400 or 800 mg) reduced craving and anxiety, and three days of CBD treatment at these doses resulted in persistent effects a week later [22]. Further, a handful of previous studies evaluating synthetic THC [23] provide signal for low dose THC to also improve withdrawal in cannabis users. Given the combined evidence of this prior work, CBD combined with low dose THC might reduce withdrawal symptoms in abstaining cannabis users and this mechanism of action may drive the therapeutic effects of hemp-derived CBD on reducing cannabis use.

This mechanism for reducing high THC use may be particularly important in a population of high potency cannabis concentrate users who report the worst symptoms of withdrawal and negative affect. However, current research is limited to synthetic or isolated forms of CBD that are not widely available. There have been no tests of the hemp-derived CBD that is widely available without a prescription across the U.S., and that comes in two forms, one with a small amount of THC (~0.3% THC, full spectrum; fsCBD) and one without THC (0% THC; broad spectrum; bsCBD). Our early data suggests CBD that also contains low levels of THC reduces THC drug reward, withdrawal, anxiety, and overall THC use in heavy concentrate users, [24] supporting the potential for hemp-derived CBD to reduce THC use and mitigate withdrawal in this high-risk group. However, no placebo-controlled trial has been conducted comparing hemp-derived CBD with and without THC on reducing THC use.

## Study objectives and hypotheses

The overarching goal of this study is to conduct a placebo-controlled RCT testing the effects of hemp-derived CBD (fsCBD vs. bsCBD vs. placebo) on reducing THC use in concentrate users with CUD. Specifically, we have the following aims:

### Aim 1

Test the effect of bsCBD (400 mg), fsCBD (400 mg), and placebo, on THC use and CUD symptoms over eight weeks of use.

#### Aim 2

Test the effect of fsCBD and bsCBD, relative to placebo, on mechanisms that may underlie their effects on reducing cannabis use, specifically affective, physiological, and physical withdrawal symptoms over the eight week trial.

#### Exploratory Aims

The study will also explore whether the effects of fsCBD and bsCBD on reducing THC use over 8 weeks are mediated through their effects on reducing affective, physiological, and physical facets of withdrawal from week 1 to week 4. In addition, we will explore whether there are sex differences in THC effects and cannabinoid metabolism.

## Methods

### Study design

**Fig 1:**
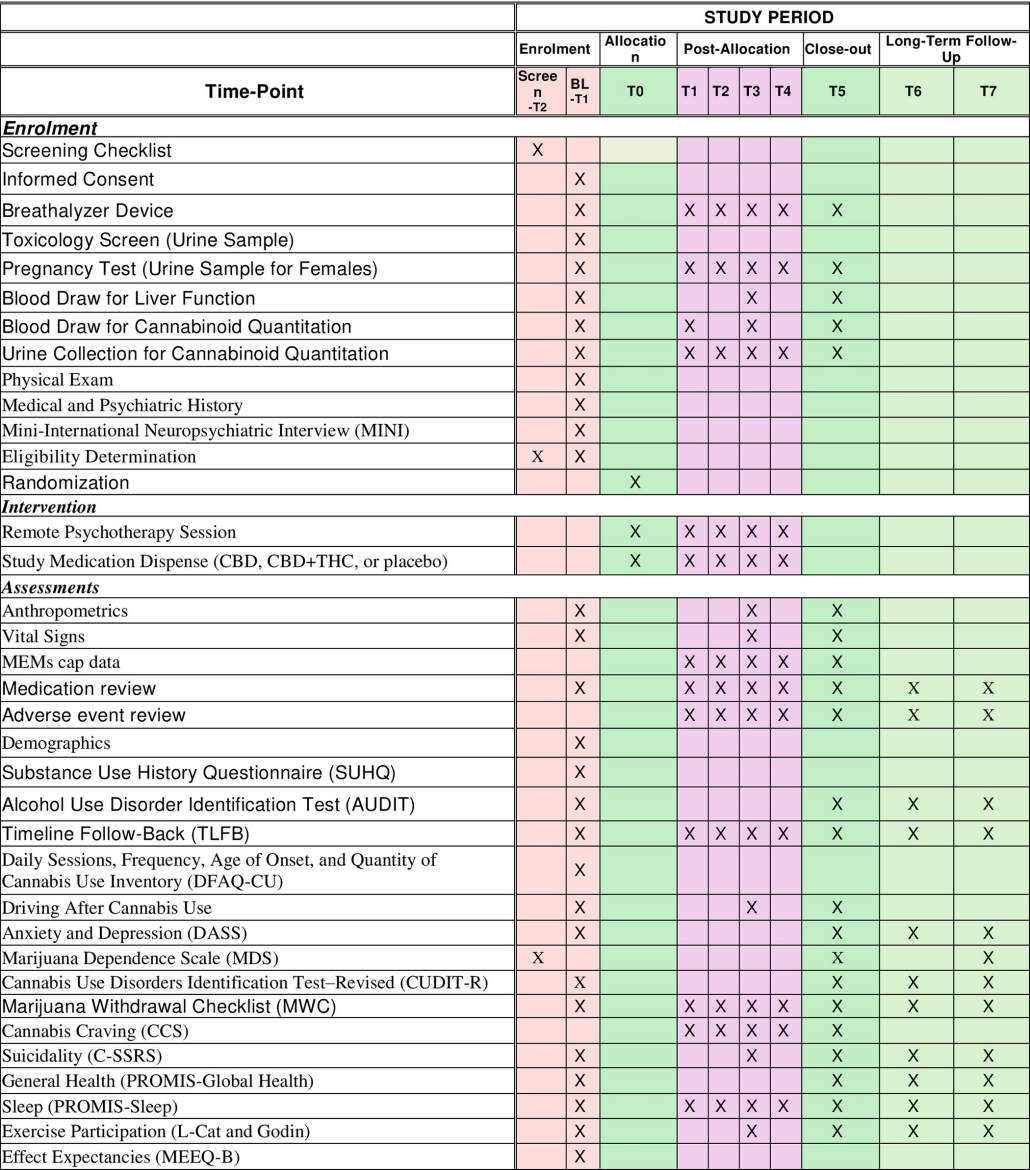
Schedule of enrollment, interventions, and assessments.

**Fig 2:**
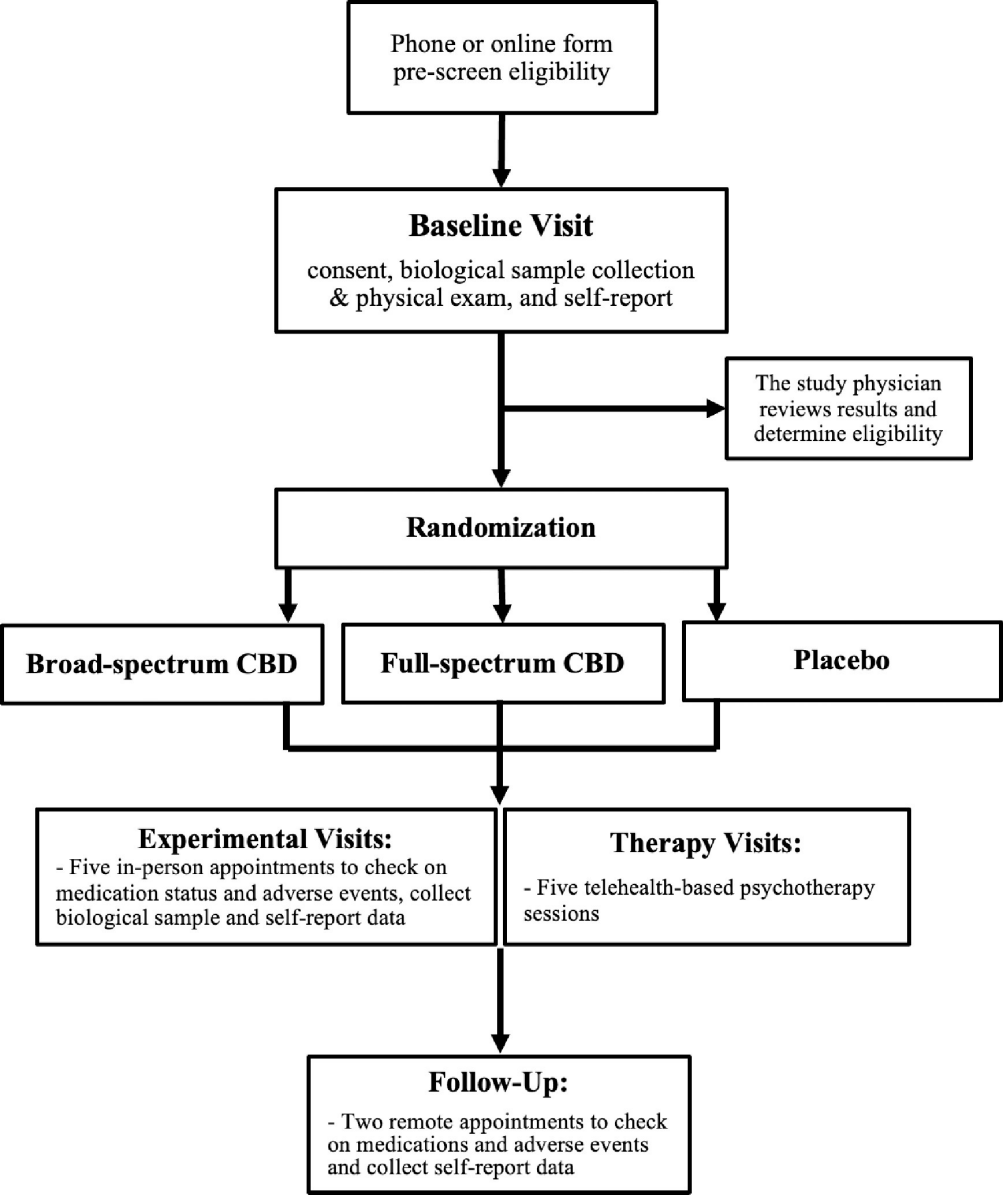
Hypothesized relationships between hemp-derived CBD and reductions in THC use via changes in withdrawal symptoms. The mediator (withdrawal) is measured part way through treatment (weeks 1 to 4), while outcome (THC use) is measured at the end of 8 weeks of treatment.

The study will be a double-blind, placebo controlled, three-arm randomized trial conducted over the course of 16 weeks (Fig 1). All participants will be concurrently enrolled in a five-session standard of care psychotherapy protocol designed to support the reduction or discontinuing of use of cannabis among individuals with at least moderate CUD. Data addressing this study’s primary and secondary aims will be collected from 8 study visits (6 in-person: Baseline, and Weeks 1, 2, 4, 6, and 8; and 2 telehealth: Weeks 12 and 16; Fig 2). For full list of protocol contributors see S1 Protocol.

### Study setting

The study will be based at the Center for Innovation and Creativity on the campus of the University of Colorado Boulder, in Boulder, CO, U.S.A. Boulder is a city of approximately 100,00 people and is part of a larger metro area encompassing Boulder, various other similarly sized municipalities, and the cities of Denver and Colorado Springs. This region is known collectively as the “Front Range Urban Corridor” and has an overall estimated population of 5 million.

### Participants & eligibility criteria

Participants will be community members who are heavy, stable concentrate users that meet criteria for at least moderate CUD and are seeking to decrease or stop cannabis use. Participants will be 21 years or older and meet all inclusion and exclusion criteria (Table 1). Participants will be pre-screened via an online eligibility form or over the phone by a trained research member. Participants meeting the inclusion/exclusion criteria will be scheduled for their baseline visit and will be instructed not to drink alcohol for 24 hours and to not use caffeine or nicotine one hour prior to the session. The study physician will review all self-report and biomarker results (e.g., liver function, counter-indicated medications and other substances) collected at the baseline visit to determine eligibility prior to participants being randomized and receiving study medication.

**Table 1:** Participants inclusion and exclusion criteria.

**Inclusion criteria**
Ages 21 and over.
Regular use (at least 4 times per week) of cannabis concentrates for at least the last year.
Meets DSM5 criteria for at least moderate CUD (4 or more symptoms).
Currently seeking to cut down or stop cannabis use.
**Exclusion criteria**
Use of any substance of abuse besides alcohol, nicotine, or cannabis (e.g., cocaine, non-prescription use of opiates, methamphetamine, MDMA, benzodiazepines, or barbiturates) in the past 90 days, as indicated by self-report and urine toxicology screening (Syva Rapid Test) at baseline.
Use of CBD-dominant products in the past 90 days, as evidenced by self-report of use of a CBD>THC product or CBD blood levels at baseline of > = 5 ng/mL which is based on the low CBD plasma levels typical of high potency concentrate users [24].*
Alcohol use on 3 or more days per week, and/or > 3 drinks per drinking day in the past 90 days. Participants must also have a breath alcohol level of 0 at the beginning of each study visit.
Daily nicotine use.
Meets DSM-5 diagnostic criteria for a psychotic disorder (e.g., schizophrenia, schizophreniform disorder, schizoaffective disorder), bipolar disorder, or major depression with suicidal ideation, or has a history of treatment for these disorders. Psychiatric disorders will be assessed with the Mini-International Neuropsychiatric Interview (MINI).
Current cardiovascular or respiratory disease (e.g., coronary artery disease, severe asthma, chronic obstructive pulmonary disease, etc.)
Current use of psychotropics (e.g., antidepressants, anxiogenics), which may dampen effects of CBD.
Currently use of anti-epileptic medications (e.g., clobazam, sodium valproate) or medications known to have major interactions with Epidiolex (buprenorphine, leflunomide, levomethadyl acetate, lomitapide, mipomersen, pexidartinib, propoxyphene, sodium oxybate, and/or teriflunomide).
Current or past hepatocellular disease, as indicated by alanine aminotransferase (ALT) or aspartate aminotransferase (AST) > 2 times the upper limit of the normal range at screening or a history of liver disease irrespective of AST and ALT at the time of screening.
For participants assigned female at birth, breastfeeding, pregnancy, or trying to become pregnant. A positive urine pregnancy test at the beginning of any study visit will result in exclusion from ongoing study participation.
History of seizures
Current use of potent CYP2C19 or CYP3A4 inducers (e.g., Rifampin, apalutamide, carbamazepine, enzalutamide, ivosidenib9, lumacaftor, ivacaftor, phenytoin, St. John’s wort, Fosphenytoin, Mitotane, Phenobarbital, Primidone), or strong CYP3A inhibitors (e.g., clarithromycin, HIV protease inhibitors, and most antifungals), 2C19 inhibitors (e.g., fluoxetine, Lansoprazole, Tricyclic antidepressants (TCAs))
Allergy to study medication ingredients (hemp seed oil, hemp extract, gelatin, glycerin)

#### Eligibility screening tools

*Clinical blood labs*. Clinical labs will include a comprehensive metabolic panel (CMP) to assess liver function and a complete blood count (CBC) to assess general health.

*Recent substance use screening tools*. A breathalyzer will ensure a breath alcohol concentration of zero at the beginning of each session. We will also conduct urine testing for recent use of drugs of abuse other than cannabis using an on-site urine toxicology test.

*Urine pregnancy*. Urine pregnancy tests will be conducted to ensure female participants are not pregnant.

### Recruitment and randomization

Participants will be recruited from the Front Range Urban Corridor area. These individuals will be recruited using flyers posted in cannabis dispensaries, advertisements on the webpages and social media pages of dispensaries and other cannabis-related businesses and organizations and social media sites, and targeted mailings. To achieve our goals for recruiting diverse participants, focused efforts will be undertaken that leverage a community engaged research approach to recruitment. Community engaged research recruitment strategies are generally more interpersonal in nature than traditional strategies, including development of content-specific presentation materials to be shared with potential partner groups and key stakeholders during community events, town halls, recurring meetings, or leadership sessions. Community-engaged recruitment also involves the planning and coordination of targeted recruitment events and follow-up with both potential participants and key stakeholders from partnering organizations. Fig 2 outlines the flow of participants through each stage of a research study.

Participants will be randomly assigned to one of three groups: broad-spectrum CBD (bsCBD: 400mg CBD and 0% THC), full-spectrum CBD (fsCBD: 400mg CBD with ~0.3% THC), or placebo (0 mg CBD and 0% THC). A pre-determined randomization table developed by the study statistician will be used and both study staff and participants will remain blind to group assignment throughout the study. A study coordinator not involved in data collection will maintain the blind.

### Intervention procedures

#### Dosing

Participants will come in person after the baseline visit and eligibility is approved by the study physician to receive medication and medication instructions. Participants in the bsCBD and fsCBD groups will take 200mg (4 x 50mg softgels) in the morning and 200mg (4 x 50mg softgels) in the evening, for a total of 400 mg/day. Participants in the placebo group will take 4 0mg CBD and 0% THC softgels in the morning and in the evening.

When the study medication and psychotherapy sessions are complete, or at any point during the study, resources will be offered and the range of continued treatment services available will be explained. If a participant wishes to pursue additional treatment, an appropriate referral will be made that day or at any future time that the participant desires.

#### Study visits and procedures

Study participants will have six in-person experimental visits on the University of Colorado Boulder campus. Self-report and biomarker collection will take place at each visit as well as medication adherence verification. Following completion of the experimental portion of the study, participants will have two telehealth visits 12 and 16 weeks following their initial baseline visit that consist of self-report measures only. To promote participant retention and completion of follow ups, participants will be compensated for completing all aspects of the study and can receive up to $390. A portion of this compensation is dependent on participant compliance to study procedures. Finally, an exit survey is conducted with all participants, regardless of completion of the study, to obtain feedback on study procedures and personnel and participant experience.

#### Five session psychotherapy

Participants in both experimental groups and the control group will participate in five telehealth-based psychotherapy sessions modified from the Substance Abuse and Mental Health Services (SAMSHA) treatment protocol to support motivation for cannabis use reduction and provide standard of care to all participants. Each psychotherapy session will take place within five days of a participant’s corresponding study visit. Study therapists will meet one-on-one with participants via Zoom during these sessions.

#### Outcome measures

*Adverse events*. Research assistants will query participants about adverse events experienced throughout the study. If an adverse event occurs, the Principal Investigator (PI) will immediately be informed and details of the event will be recorded in an Adverse Event Log for event reporting. In addition, the Study Physician will review all adverse events to determine the relationship of the event to the study intervention, the severity of the adverse event, and the expectedness of the adverse events.

The Study Physician and participants will be blinded to condition assignment, however, the blind will be broken in emergencies when knowledge of the participant’s group in necessary for their safety. Following an adverse event or other safety concern, the participant may be instructed to discontinue the intervention if it is in the best interest of the participant in opinion of the PI (a licensed clinical psychologist) or Study Physician. The participant will be made aware of compensation policies for research related injury during the informed consent procedure.

*Anthropometrics*. Height and weight will be measured with a stadiometer and scale. Waist circumference and hip circumference will be measured with a measuring tape.

*Anxiety*, *depression*, *and suicidality*. The Depression Anxiety Stress Scale (DASS) [25] consists of 21 items designed to measure the emotional states of depression, anxiety, and stress in three subscales. The Columbia-Suicide Severity Rating Scale (C-SSRS) [26] includes six items that categorizes an individual as low, moderate, or high risk for suicidality.

*CUD*, *cannabis withdrawal symptoms*, *and cannabis craving*. The Marijuana Dependence Scale (MDS) [27] is based on DSM V criteria that were converted to a self-report measure. Individuals report on each dependence item and items are then summed to form the scale (α = .85). This scale is validated and has been previously used in the cannabis literature. The Cannabis Use Disorders Identification Test–Revised (CUDIT-R) [28] consists of 8 items designed to identify potentially problematic or harmful recent cannabis use. The Marijuana Withdrawal Checklist (MWC) [29], will assess 15 cannabis withdrawal symptoms. The Cannabis Craving Scale will assess cannabis craving [30].

*Demographics*. Age, sex assigned at birth, gender identity, sexual orientation, marital status, race and ethnicity, socioeconomic status, occupation/retirement status, income, education, and neighborhood (zip code and county) will be assessed.

*Effect expectancies*. To assess and control for differences in cannabis effect expectancies between participants, participants will complete the Marijuana Effect Expectancy Questionnaire—Brief [31] and the Cannabis Effect Expectancy–Medical (CEEQ-M) [32].

*Exercise participation and general health*. The Stanford Leisure-Time Activity Categorical Item (L-Cat) [33] and the Godin Leisure-Time Exercise Questionnaire [34] will be used to quantify exercise behavior. NIH’s Patient Reported Outcomes Measurement Information System (PROMIS) Global Health survey [35] includes 10 questions to assess an individual’s physical, mental, and social health.

*Medical and psychiatric history*. A physician will ask participants about their medical and psychiatric history. In addition, research assistants will conduct the Mini-International Neuropsychiatric Interview (MINI) [36] to assess CUD diagnostic criteria and psychiatric comorbidity. The PI will supervise the training of research assistants in the conduct of the MINI to ensure competency.

*Medication adherence*. Medication Event Monitory System (MEMS) caps will be used to collect data about frequency, time, and date of bottle openings. Manual softgel counts will also be conducted.

*Physical exam and vital signs*. Heart rate, blood pressure, and oximetry on room air will be measured. A physical exam will be conducted by a physician to assess general health.

*Self-report measures of substance use*. A Substance Use History Questionnaire (SUHQ) will assess frequency of lifetime and recent illicit drug use. Current medications will also be tracked. The Daily Sessions, Frequency, Age of Onset, and Quantity of Cannabis Use Inventory (DFAQ-CU) [37] will be used to collect information on the frequency and quantity of cannabis use, age of first use, peer use, perceived risk from cannabis, and perceived availability of cannabis. The Alcohol Use Disorder Identification Test (AUDIT) [38] will be used to examine the extent of alcohol use and problems related to alcohol use. A Timeline Follow-Back (TLFB) [39] will be used to assess daily substance use. The TLFB is a calendar assisted tool that provides the subject with temporal cues to increase the accuracy of recall. This instrument has demonstrated test-retest reliability and validity [40]. The TLFB also records alcohol use, tobacco use, use of illegal drugs, and recreational use of prescription drugs such as anti-depressants, Adderall, Ritalin, and Vicodin. We have modified our TLFB procedure to estimate in detail the frequency, type, amount, and potency of cannabis use each day [41]. Participants will also be asked a question about frequency of Driving After Cannabis Use.

*Sleep*. NIH’s Patient Reported Outcomes Measurement Information System (PROMIS) Sleep Disturbance and Sleep-Related Impairment measure [42] will assess sleep functioning.

*THC exposure*. Each blood and urine sample will be assayed using HPLC-MS-MS for cannabinoids including THC and THC-COOH (inter-assay precision is within 85–115% and total imprecision, except at lower limit of quantification, is better than 15%). The cannabinoid assays utilized in the iC42 laboratory have been completely validated following FDA guidelines for bioanalytical method development, and the methods are published [43].

### Data quality control

All of the measures used in the study have been validated in the literature with the exception of the single item driving question. All scales will be additionally evaluated for internal consistency using Cronbach’s Alpha. Research personnel will be thoroughly trained on study procedures including verification of competency by “mock” visit. Research study encounters will be tracked using study procedure checklists. Research supervisors will oversee the data collection process and hold weekly meetings with all research staff. Research data management professionals will oversee data storage and validation. Psychotherapy sessions will be documented using session checklists and therapists will hold biweekly clinical supervision and fidelity meetings with a PhD level licensed clinical psychologist.

All coded data will be entered into a centralized REDCap data management system either directly by participants or through secure automated data transfer. This project will only be accessible by research staff via their personal REDCap credentials, and only the PI will have “user rights” to assign team members to study related projects. The results of clinical blood tests (CMP and CBC) will be made available to participants upon request. Any clinically relevant research results (e.g., abnormal CMP or CBC) will also be disclosed to participants.

### Statistical approach

#### Power analyses

Our approach to power analysis and accounting for attrition is extremely conservative, in that the techniques we will utilize in data analysis use state of the art recommendations in iterating the estimation of missing data [44]. All analyses are intent-to-treat and all available data are utilized. Since power is determined by the analysis that requires the largest number of participants (Exploratory Aim 3), we based our power analysis on the mediational model proposed in Exploratory Aim 3 (Fig 2). We expected small to moderate coefficients for most paths in the mediational models, with parameter estimates in the range of .30 to .35 for paths from the active medication versus placebo contrast to the mediators and from the withdrawal mediators to THC use. We estimated somewhat smaller parameter estimates of .25 from the fsCBD vs bsCBD contrast to the mediators. Power analyses were conducted in Mplus and then in SAS following procedures for estimating the power of the likelihood ratio test of the significance of parameters in structural equation models [45].

We utilized Monte Carlo simulation to generate a population covariance matrix based on the hypothesized parameters in the model. We evaluated power at a range of sample sizes. For the smallest path coefficient in the model, i.e., that between the fsCBD vs bsCBD contrast to the mediators, assuming two-tailed alpha of .05, a sample size of 100 gave us only .63 power, a sample size of 125 gave us .73 power and at sample size of 150 we have .80 power. For each of the larger paths in the model, we have over .91 power with 150 participants. Based on our previous studies with this population, we anticipate a 10% attrition rate between the baseline session and the week 8 session. Thus, we will recruit a total of 55 per medication group (n = 165) with a target of n = 150 complete participants.

#### Statistical analysis plan

Analyses will be conducted with R (R Core Team, 2020). All aims will be tested with a multilevel modeling framework to account for the repeated observations nested within participants over time. Preliminary analyses will evaluate all data for normality and reliability. Three-group ANOVAs and χ^2^ tests will be used to compare baseline variables between groups.

### Funding, ethical considerations & pre-registration

This study is funded by a grant to the University of Colorado Boulder (1 R01 DA059234) from the National Institute on Drug Abuse (NIDA). This study was pre-registered on clinicaltrials.gov (NCT06107062). All recruited participants will be engaged in an informed consent process with study personnel to ensure they are aware of the study goals, the benefits, any possible risks related to the study medication and their participation. To protect the privacy and confidentiality of study participants, screening, the informed consent process, and all subsequent study assessments, including blood draws, will be conducted within private rooms. At the end of the study, all links between participant name and coding number will be destroyed, at which point the data will be considered de-identified. All other records will continue to be kept in a secure location for as long a period as dictated by IRB, Institutional, and sponsor regulations and requirements.

## Discussion

Extant data indicate that cannabis concentrate use is rapidly increasing and that heavy THC exposure leads to greater cannabis-related harms, including more cannabis use, withdrawal, and CUD symptoms. While the field currently has no existing medication to treat CUD, the non-intoxicating cannabinoid CBD shows promise as a candidate CUD medication and may reduce cannabis use and withdrawal, especially if higher doses and/or hemp-derived fsCBD are used. Considering the emerging signals from clinical trials and the wide availability of bs/fsCBD on legal markets, this project aims to extend these findings to test 400 mg of a widely available plant-derived CBD formulation with and without THC in a rigorous RCT framework on reducing THC use and withdrawal in understudied group of high potency concentrate users.

## Supporting information

S1 Checklist Human participants research checklist. (DOCX)

S2 Checklist SPIRIT 2013 checklist: Recommended items to address in a clinical trial protocol and related documents*. (DOC)

S1 File (PDF)

S2 File (PDF)

S3 File (PDF)

S4 File (PDF)

S5 File (PDF)

S1 Protocol (PDF)
