# Supplementary material for: LOTUS: Protocol for a double-blind placebo controlled randomized trial of hemp-derived cannabidiol for the treatment of cannabis use disorder
Source: PLoS One. 2024 Sep 30;19(9):e0308262. doi: 10.1371/journal.pone.0308262 (PMC11441658; doi:10.1371/journal.pone.0308262)
Supplement: S1 File — (PDF) [file pone.0308262.s003.pdf]

Department of Health and Human Services
National Institutes of Health
NATIONAL INSTITUTE ON DRUG ABUSE

Notice of Award
FAIN# R01DA059234
Federal Award Date
09/08/2023

Recipient Information

1. Recipient Name
THE REGENTS OF THE UNIVERSITY OF COLORADO
3100 MARINE ST
STE 481 572 UCB
BOULDER, CO 80309

2. Congressional District of Recipient
02

3. Payment System Identifier (ID)
1846000555A2

4. Employer Identification Number (EIN)
846000555

5. Data Universal Numbering System (DUNS)
007431505

6. Recipient's Unique Entity Identifier
SPVKK1RC2MZ3

7. Project Director or Principal Investigator
L. Cinnamon Bidwell, PHD (Contact)
Assistant Professor
lcb@colorado.edu
781-608-3711

8. Authorized Official
Tessa Brandt
tessa.brandt@colorado.edu
303-735-8462

Federal Agency Information

9. Awarding Agency Contact Information
Pamela G. Fleming
Grants Management Officer
NATIONAL INSTITUTE ON DRUG ABUSE
pfleming@mail.nih.gov
301-480-1159

10. Program Official Contact Information
Aidan Hampson
Progam Official
NATIONAL INSTITUTE ON DRUG ABUSE
aidan.hampson@nih.gov
301-827-5925

Federal Award Information

11. Award Number
1R01DA059234-01A1

12. Unique Federal Award Identification Number (FAIN)
R01DA059234

13. Statutory Authority
42 USC 241 42 CFR 52

14. Federal Award Project Title
Hemp-derived Cannabidiol for the treatment of cannabis use disorder in concentrate users: A double-blind placebo-controlled randomized trial

15. Assistance Listing Number
93.279

16. Assistance Listing Program Title
Drug Abuse and Addiction Research Programs

17. Award Action Type
New Competing

18. Is the Award R&D?
Yes

Summary Federal Award Financial Information

**19. Budget Period Start Date** 09/15/2023 **– End Date** 07/31/2024	
**20. Total Amount of Federal Funds Obligated by this Action**	$741,850
20 a. Direct Cost Amount	$493,913
20 b. Indirect Cost Amount	$247,937
**21.** Authorized Carryover	
**22.** Offset	
**23.** Total Amount of Federal Funds Obligated this budget period	$741,850
**24. Total Approved Cost Sharing or Matching, where applicable**	$0
**25. Total Federal and Non-Federal Approved this Budget Period**	$741,850
**26. Project Period Start Date** 09/15/2023 **– End Date** 07/31/2028	
**27.** Total Amount of the Federal Award including Approved Cost Sharing or Matching this Project Period	$741,850

28. Authorized Treatment of Program Income
Additional Costs

29. Grants Management Officer - Signature
Carol Alderson

30. Remarks
Acceptance of this award, including the "Terms and Conditions," is acknowledged by the recipient when funds are drawn down or otherwise requested from the grant payment system.

RESEARCH
Department of Health and Human Services
National Institutes of Health

NATIONAL INSTITUTE ON DRUG ABUSE

SECTION I – AWARD DATA – 1R01DA059234-01A1

Principal Investigator(s):
L. Cinnamon Bidwell (contact), PHD
KENT E. HUTCHISON, PHD

Award e-mailed to: ocg@colorado.edu

Dear Authorized Official:

The National Institutes of Health hereby awards a grant in the amount of $741,850 (see "Award Calculation" in Section I and "Terms and Conditions" in Section III) to UNIVERSITY OF COLORADO in support of the above referenced project. This award is pursuant to the authority of 42 USC 241 42 CFR 52 and is subject to the requirements of this statute and regulation and of other referenced, incorporated or attached terms and conditions.

Acceptance of this award, including the "Terms and Conditions," is acknowledged by the recipient when funds are drawn down or otherwise requested from the grant payment system.

Each publication, press release, or other document about research supported by an NIH award must include an acknowledgment of NIH award support and a disclaimer such as "Research reported in this publication was supported by the National Institute On Drug Abuse of the National Institutes of Health under Award Number R01DA059234. The content is solely the responsibility of the authors and does not necessarily represent the official views of the National Institutes of Health." Prior to issuing a press release concerning the outcome of this research, please notify the NIH awarding IC in advance to allow for coordination.

Award recipients must promote objectivity in research by establishing standards that provide a reasonable expectation that the design, conduct and reporting of research funded under NIH awards will be free from bias resulting from an Investigator's Financial Conflict of Interest (FCOI), in accordance with the 2011 revised regulation at 42 CFR Part 50 Subpart F. The Institution shall submit all FCOI reports to the NIH through the eRA Commons FCOI Module. The regulation does not apply to Phase I Small Business Innovative Research (SBIR) and Small Business Technology Transfer (STTR) awards. Consult the NIH website http://grants.nih.gov/grants/policy/coi/ for a link to the regulation and additional important information.

If you have any questions about this award, please direct questions to the Federal Agency contacts.

Sincerely yours,

Carol Alderson
Grants Management Officer
NATIONAL INSTITUTE ON DRUG ABUSE

Additional information follows

Cumulative Award Calculations for this Budget Period (U.S. Dollars)

**Salaries and Wages**	$237,798
**Fringe Benefits**	$78,108
**Personnel Costs (Subtotal)**	$315,906
**Materials & Supplies**	$10,374
**Other**	$109,696
**Subawards/Consortium/Contractual Costs**	$55,087
**ADP/Computer Services**	$2,850

**Federal Direct Costs**	$493,913
**Federal F&A Costs**	$247,937
**Approved Budget**	$741,850
**Total Amount of Federal Funds Authorized (Federal Share)**	$741,850
**TOTAL FEDERAL AWARD AMOUNT**	$741,850
**AMOUNT OF THIS ACTION (FEDERAL SHARE)**	$741,850

**SUMMARY TOTALS FOR ALL YEARS** (for this Document Number)		
**YR**	**THIS AWARD**	**CUMULATIVE TOTALS**
1	$741,850	$741,850
2	$725,910	$725,910
3	$710,354	$710,354
4	$694,985	$694,985
5	$674,798	$674,798

Recommended future year total cost support, subject to the availability of funds and satisfactory progress of the project

Fiscal Information:

Payment System Identifier: 1846000555A2
Document Number: RDA059234A
PMS Account Type: P (Subaccount)
Fiscal Year: 2023

IC	CAN	2023	2024	2025	2026	2027
DA	8472630	$741,850	$725,910	$710,354	$694,985	$674,798

Recommended future year total cost support, subject to the availability of funds and satisfactory progress of the project

NIH Administrative Data:
PCC: CC/AJH / **OC**: 41021 / **Released**: Alderson, Carol 08/30/2023
Award Processed: 09/08/2023 12:21:29 AM

SECTION II – PAYMENT/HOTLINE INFORMATION – 1R01DA059234-01A1

For payment and HHS Office of Inspector General Hotline information, see the NIH Home Page at http://grants.nih.gov/grants/policy/awardconditions.htm

SECTION III – STANDARD TERMS AND CONDITIONS – 1R01DA059234-01A1

This award is based on the application submitted to, and as approved by, NIH on the above-titled project and is subject to the terms and conditions incorporated either directly or by reference in the following:

a. The grant program legislation and program regulation cited in this Notice of Award.
b. Conditions on activities and expenditure of funds in other statutory requirements, such as those included in appropriations acts.
c. 45 CFR Part 75.
d. National Policy Requirements and all other requirements described in the NIH Grants Policy Statement, including addenda in effect as of the beginning date of the budget period.
e. Federal Award Performance Goals: As required by the periodic report in the RPPR or in the final progress report when applicable.
f. This award notice, INCLUDING THE TERMS AND CONDITIONS CITED BELOW.

(See NIH Home Page at http://grants.nih.gov/grants/policy/awardconditions.htm for certain references cited above.)

Research and Development (R&D): All awards issued by the National Institutes of Health (NIH) meet the definition of “Research and Development” at 45 CFR Part§ 75.2**.** As such, auditees should identify NIH awards as part of the R&D cluster on the Schedule of Expenditures of Federal Awards (SEFA). The auditor should test NIH awards for compliance as instructed in Part V, Clusters of Programs. NIH recognizes that some awards may have another classification for purposes of indirect costs. The auditor is not required to report the disconnect (i.e., the award is classified as R&D for Federal Audit Requirement purposes but non-research for indirect cost rate purposes), unless the auditee is charging indirect costs at a rate other than the rate(s) specified in the award document(s).

An unobligated balance may be carried over into the next budget period without Grants Management Officer prior approval.

This grant is subject to Streamlined Noncompeting Award Procedures (SNAP).

This award is subject to the requirements of 2 CFR Part 25 for institutions to obtain a unique entity identifier (UEI) and maintain an active registration in the System for Award Management (SAM). Should a consortium/subaward be issued under this award, a UEI requirement must be included. See http://grants.nih.gov/grants/policy/awardconditions.htm for the full NIH award term implementing this requirement and other additional information.

This award has been assigned the Federal Award Identification Number (FAIN) R01DA059234. Recipients must document the assigned FAIN on each consortium/subaward issued under this award.

Based on the project period start date of this project, this award is likely subject to the Transparency Act subaward and executive compensation reporting requirement of 2 CFR Part 170. There are conditions that may exclude this award; see http://grants.nih.gov/grants/policy/awardconditions.htm for additional award applicability information.

In accordance with P.L. 110-161, compliance with the NIH Public Access Policy is now mandatory. For more information, see NOT-OD-08-033 and the Public Access website: http://publicaccess.nih.gov/.

This award provides support for one or more clinical trials. By law (Title VIII, Section 801 of Public Law 110-85), the “responsible party” must register “applicable clinical trials” on the ClinicalTrials.gov Protocol Registration System Information Website. NIH encourages registration of all trials whether required under the law or not. For more information, see http://grants.nih.gov/ClinicalTrials_fdaaa/

Recipients must administer the project in compliance with federal civil rights laws that prohibit discrimination on the basis of race, color, national origin, disability, age, and comply with applicable conscience protections. The recipient will comply with applicable laws that prohibit discrimination on the

basis of sex, which includes discrimination on the basis of gender identity, sexual orientation, and pregnancy. Compliance with these laws requires taking reasonable steps to provide meaningful access to persons with limited English proficiency and providing programs that are accessible to and usable by persons with disabilities. The HHS Office for Civil Rights provides guidance on complying with civil rights laws enforced by HHS. See https://www.hhs.gov/civil-rights/for-providers/provider-obligations/index.html and https://www.hhs.gov/.

- Recipients of FFA must ensure that their programs are accessible to persons with limited English proficiency. For guidance on meeting the legal obligation to take reasonable steps to ensure meaningful access to programs or activities by limited English proficient individuals, see https://www.hhs.gov/civil-rights/for-individuals/special-topics/limited-english-proficiency/fact-sheet-guidance/index.html and https://www.lep.gov.
- For information on an institution's specific legal obligations for serving qualified individuals with disabilities, including providing program access, reasonable modifications, and to provide effective communication, see http://www.hhs.gov/ocr/civilrights/understanding/disability/index.html.
- HHS funded health and education programs must be administered in an environment free of sexual harassment; see https://www.hhs.gov/civil-rights/for-individuals/sex-discrimination/index.html. For information about NIH's commitment to supporting a safe and respectful work environment, who to contact with questions or concerns, and what NIH's expectations are for institutions and the individuals supported on NIH-funded awards, please see https://grants.nih.gov/grants/policy/harassment.htm.
- For guidance on administering programs in compliance with applicable federal religious nondiscrimination laws and applicable federal conscience protection and associated anti-discrimination laws, see https://www.hhs.gov/conscience/conscience-protections/index.html and https://www.hhs.gov/conscience/religious-freedom/index.html.

In accordance with the regulatory requirements provided at 45 CFR 75.113 and Appendix XII to 45 CFR Part 75, recipients that have currently active Federal grants, cooperative agreements, and procurement contracts with cumulative total value greater than $10,000,000 must report and maintain information in the System for Award Management (SAM) about civil, criminal, and administrative proceedings in connection with the award or performance of a Federal award that reached final disposition within the most recent five-year period. The recipient must also make semiannual disclosures regarding such proceedings. Proceedings information will be made publicly available in the designated integrity and performance system (currently the Federal Awardee Performance and Integrity Information System (FAPIIS)). Full reporting requirements and procedures are found in Appendix XII to 45 CFR Part 75. This term does not apply to NIH fellowships.

Treatment of Program Income:
Additional Costs

SECTION IV – DA SPECIFIC AWARD CONDITIONS – 1R01DA059234-01A1

Clinical Trial Indicator: Yes
This award supports one or more NIH-defined Clinical Trials. See the NIH Grants Policy Statement Section 1.2 for NIH definition of Clinical Trial.

This award contains grant-specific restrictions. These restrictions may only be lifted by a revised Notice of Award (NoA).

RESTRICTED AWARD
No funds may be expended for this grant until NIDA has received notice that -

1) Thirty days have elapsed since issue of the Food and Drug Administration (FDA) acknowledgement of receipt of the Investigational New Drugs (INDs) associated with both the full spectrum and broad-spectrum hemp formulations to be used in this study

2) During the 30-day period following FDA acknowledgement of receipt, no clinical hold, or any request from FDA to extend the FDA evaluation period has been received pertaining to the acceptability of the IND submissions.

A revised Notice of Award (NoA) will be issued, once this has occurred to lift the restriction on the funds, accordingly.

HUMAN SUBJECTS RESTRICTED- IRB APPROVAL REQUIRED
This award is issued without a currently valid certification of Institutional Review Board (IRB) approval for this project with the following restriction: Only activities that are clearly severable and independent from activities that involve human subjects may be conducted pending NIDA's acceptance of the certification of IRB approval. No funds may be drawn down from the payment system and no obligations may be made against Federal funds for research involving human subjects at any site engaged in such research for any period not covered by an Office for Human Research Protections Assurance and an IRB approval consistent with the requirements of 45 CFR Part 46. **IRB approval must be submitted within 60 days from the issue date of the Notice of Award**.

See the NIH Grants Policy Statement, section on Human Subjects Protections http://grants.nih.gov/policy/nihgps/index.htm for specific requirements related to the protection of human subjects, which are applicable to this term and condition of award.

HUMAN SUBJECTS RESTRICTION- DATA AND SAFETY MONITORING PLAN REQUIRED
This award is contingent upon receipt and written approval, **within 60 days from the issue date of the Notice of Award**, by the NIDA Program Official of the detailed Data and Safety Monitoring Plan (DSMP) for the trial(s) to be conducted under this award. The elements of the DSMP are delineated in the DSMP guidelines provided by NIH and NIDA, respectively. The Authorized Organizational Representative should submit the DSMP to the NIDA Program Official and Grants Management Specialist listed in eRA Commons. Recruitment of subjects for the trial supported by this award cannot start until the NIDA Program Official approves the DSMP and a revised Notice of Award is issued to remove the restriction.

FY2023 FUNDING
The award amount for the current year is based upon IRG/Council recommendations, cost analysis, program priorities and availability of funds.

BUDGET PERIOD
Future year anniversary dates for this grant will be August 1 and the Research Performance Progress Report (RPPR) is due on June 15.

MULTIPLE PI
The following principal investigators (PIs) are associated with this project:

- L. Cinnamon Bidwell, University of Colorado,
- Kent E. Hutchinson, University of Colorado, Anschutz

Dr. Bidwell is the contact PI for correspondence purposes. As this grant has multiple PIs, all NIH Grant policies regarding multiple PIs, including, but, not limited to all prior approval requirements, must be followed accordingly, see NIH Grants Policy Statement, section on Multiple Program Director/Principal Investigators, found at http://grants.nih.gov/policy/nihgps/index.htm.

CONSORTIUM ACTIVITY
This award includes funds awarded for consortium activities. Consortia are to be established and administered as described in the NIH Grants Policy Statement (NIH GPS), Section on Consortium Agreements - http://grants.nih.gov/policy/nihgps/index.htm.

Version: 13 - 8/3/2022 12:59 PM | Generated on: 9/8/2023 12:21 AM

The recipient is reminded of the *NIH Updated Policy Guidance for Subaward/Consortium Written Agreements* as published in the NIH Guide notice NOT-OD-23-133 on May 19, 2023 with an effective date of October 1, 2023. The notice can be found here: https://grants.nih.gov/grants/guide/notice-files/NOT-OD-23-133.html

NIH SALARY CAP
None of the funds in this award shall be used to pay the salary of an individual at a rate in excess of the current salary cap. Therefore, this budget period and/or future budgeted years are adjusted accordingly, if applicable. Current salary cap levels can be found at the following URL: http://grants2.nih.gov/grants/policy/salcap_summary.htm.

NIDA TERMS
In conjunction with the Acknowledgment of Federal Funding Requirement (as specified in the NIH Grants Policy Statement, Appropriation Mandates http://grants.nih.gov/policy/nihgps/index.htm), in order to most effectively disseminate research results, advance notice should be given to NIDA that research findings are about to be published so that we may coordinate accurate and timely release to the media. This information will be embargoed until the publication date. Please see the NIDA Special Considerations Page for guidance on coordination with the NIDA Press Office at https://www.drugabuse.gov/funding/special-considerations-for-nida-funding, or contact the NIDA Press Office at media@nida.nih.gov.

Please see Special Considerations for NIDA Funding Opportunities and Awards at https://www.drugabuse.gov/funding/special-considerations-for-nida-funding.

Data Management and Sharing Policy: Applicable

This project is expected to generate scientific data. Therefore, the Final NIH Policy for Data Management and Sharing applies. The approved Data Management and Sharing (DMS) Plan is hereby incorporated as a term and condition of award, and the recipient shall manage and disseminate scientific data in accordance with the approved plan. Any significant changes to the DMS Plan (e.g., new scientific direction, a different data repository, or a timeline revision) require NIH prior approval. Failure to comply with the approved DMS plan may result in suspension and/or termination of this award, withholding of support, audit disallowances, and/or other appropriate action. See NIH Grants Policy Statement Section 8.2.3 for more information on data management and sharing expectations.

Budget	Year 1	Year 2	Year 3	Year 4	Year 5
DMS Costs	$9,207	$9,536	$9,878	$10,233	$10,601

SPREADSHEET SUMMARY
AWARD NUMBER: 1R01DA059234-01A1

INSTITUTION: UNIVERSITY OF COLORADO

Budget	Year 1	Year 2	Year 3	Year 4	Year 5
Salaries and Wages	$237,798	$237,798	$229,862	$222,537	$235,356
Fringe Benefits	$78,108	$78,108	$75,040	$72,208	$77,164
Personnel Costs (Subtotal)	$315,906	$315,906	$304,902	$294,745	$312,520
Materials & Supplies	$10,374	$10,374	$7,192	$7,192	$5,909
Travel		$5,455	$7,476	$7,476	$7,476
Other	$109,696	$92,630	$93,430	$93,767	$64,376
Subawards/Consortium/Contractual Costs	$55,087	$55,088	$55,088	$55,088	$55,088
Publication Costs		$1,425	$2,850	$2,850	$2,850

Version: 13 - 8/3/2022 12:59 PM | Generated on: 9/8/2023 12:21 AM

ADP/Computer Services	$2,850	$2,850	$2,850	$2,850	$2,850
TOTAL FEDERAL DC	$493,913	$483,728	$473,788	$463,968	$451,069
TOTAL FEDERAL F&A	$247,937	$242,182	$236,566	$231,017	$223,729
TOTAL COST	$741,850	$725,910	$710,354	$694,985	$674,798

Facilities and Administrative Costs	Year 1	Year 2	Year 3	Year 4	Year 5
F&A Cost Rate 1	56.5%	56.5%	56.5%	56.5%	56.5%
F&A Cost Base 1	$438,826	$428,640	$418,700	$408,880	$395,981
F&A Costs 1	$247,937	$242,182	$236,566	$231,017	$223,729
