# Supplementary material for: LOTUS: Protocol for a double-blind placebo controlled randomized trial of hemp-derived cannabidiol for the treatment of cannabis use disorder
Source: PLoS One. 2024 Sep 30;19(9):e0308262. doi: 10.1371/journal.pone.0308262 (PMC11441658; doi:10.1371/journal.pone.0308262)
Supplement: S2 File — (PDF) [file pone.0308262.s004.pdf]

Institutional Review Board
563 UCB
Boulder, CO 80309
Phone: 303.735.3702
Fax: 303.735.5185
FWA: 00003492

INSTITUTIONAL REVIEW BOARD

APPROVAL

18-Apr-2024

Dear Lorna Bidwell Kovalev,

On 18-Apr-2024 the IRB reviewed the following protocol:

Type of Submission:	Amendment
Review Category:	Expedited
Risk Level:	Minimal
Title:	Hemp-derived cannabidiol for the treatment of cannabis use disorder: A double-blind placebo-controlled randomized trial
Investigator:	Bidwell Kovalev, Lorna
Protocol #:	23-0373
Funding:	Federal
IND or IDE:	IND present
Documents Approved:	23-0373 Protocol (IRBAppvd 04.18.24); 23-0373 Surveys (IRBAppvd 04.18.24); 23-0373 Consent (IRBAppvd 04.18.24);
Documents Reviewed:	Lab Management for Determining and Reporting Adverse Events v03.25.24.docx; Dey CITI GCP for Clinical Trials w Invest Drugs and Med Devices.pdf; GM_GCP.pdf; Natal_FDAFocusCITI.pdf; JW_GCP.pdf; NM GCP FDA.pdf; HRP-213 Amendment-v5;
Description:	1. The following questions/measures have been added:<br>a. Social determinants of health / Neighborhood (zip code and county questions)<br>b. PROMIS Global Health measure<br>c. Driving After Cannabis Use Question<br>d. Cannabis Craving Scale<br>2. The following measures have been removed:<br>a. Beck Anxiety Inventory<br>b. Beck Depression Index – II<br>c. Health-Related Quality of Life Short Form<br>3. Blood assessment of THC has been removed from the Week 2 and Week 6 visits.<br>4. The compensation for the in-person visits has been updated so that participants receive $40 for the Week 1, 2, and 6 visits, and $60 for the Week 4 and 8 visits.<br>5. Compensation has been added for completion of the daily diaries. Participants will receive $15 in cash for completing at least 80% (23 of 28) of daily diaries between the Week 1 and 4 visits, and $15 in cash for completing at least 80% (23 of 28) of daily diaries between the Week 4 and 8 visits.<br>6. The daily diary survey has been updated. A question about nicotine use and a question about cannabis craving has been added. The visual of cannabis flower has been removed and the question about cannabis has been updated.

	7. The word “marijuana” has been replaced with “cannabis” in the MEEQ-B survey.<br>8. The informed consent section has been updated to reflect that it will be obtained at the Clinical Translational Research Center.<br>9. The blood draw volumes on the consent form have been updated.<br>10. A detailed description of adverse event determination and reporting has been provided in the document (“Lab Management for Determining and Reporting Adverse Events”).<br>11. Added study staff.

IRB Approval for this protocol will expire on **13-Dec-2024**.

You are required to use the IRB Approved versions of study documents to conduct your research. The IRB Approved documents can be found here: Approved Documents

In conducting this protocol you must follow the requirements listed in the INVESTIGATOR MANUAL (HRP-103).

Sincerely,
Misty White
IRB Manager
Institutional Review Board

This letter has been electronically signed in accordance with all applicable regulations, and a copy is retained within the University of Colorado Boulder’s IRB records.
