# Supplementary material for: LOTUS: Protocol for a double-blind placebo controlled randomized trial of hemp-derived cannabidiol for the treatment of cannabis use disorder
Source: PLoS One. 2024 Sep 30;19(9):e0308262. doi: 10.1371/journal.pone.0308262 (PMC11441658; doi:10.1371/journal.pone.0308262)
Supplement: S3 File — (PDF) [file pone.0308262.s005.pdf]

Title of research study: Hemp-derived cannabidiol for the treatment of cannabis use disorder: A double-blind placebo-controlled randomized trial

IRB Protocol Number: 23-0373

Investigator: L. Cinnamon Bidwell, PhD

Sponsor: National Institute on Drug Abuse

Key Information

- The purpose of the study is to better understand the effects of hemp-derived cannabidiol (CBD) with and without trace amounts of ∆9-tetrahydrocannabinol (THC) (<0.3%), on reducing THC use and cannabis use disorder (CUD) symptoms in cannabis concentrate users. CBD and THC are two different substances called cannabinoids found in cannabis.
- You will be asked to complete seven in-person visits, five virtual therapy sessions, and two virtual follow-up visits over the course of 16 weeks. The therapy sessions will focus on reducing cannabis use. The total amount of time to complete the study is 13.5 hours.
- You will be asked to take four softgels in the morning and four softgels in the evening for a total of eight softgels per day for 8 weeks. The softgels you take will either have CBD with trace amounts of THC, CBD only, or Hemp Seed Oil, which does not contain any CBD or THC. The kind of softgels that you take will be chosen by chance, like flipping a coin. You should take the softgels with food. You should keep the softgels in a safe place away from other people, especially children.
- You will be asked to refrain from alcohol use for 24 hours and caffeine and nicotine use for 1 hour before each visit.
- The study medication is not FDA-approved and potential side effects include drowsiness, gastrointestinal discomfort, and changes in liver function.

Purpose of the Study

Because of the high amounts of THC found in cannabis concentrates, use of these products can cause greater cannabis-related harms than other products, including more cannabis use, withdrawal, and CUD symptoms. While there are currently no existing medications to treat

CUD, the non-intoxicating cannabinoid CBD shows promise for reducing THC use and withdrawal. This study will compare the effects of hemp-derived CBD with and without THC on reducing THC use and withdrawal using hemp-derived CBD products as a Food and Drug Administration (FDA) Investigational New Drug (IND).

We expect that you will be in this research study for a total of 16 weeks. The total amount of time you spend completing study tasks will be approximately 13.5 hours.

We expect about 165 people will be in this research study.

Explanation of Procedures

If you join the study, you will be asked to participate in seven in-person visits and five virtual therapy sessions over the course of 8 weeks. All in-person visits will take place in the morning. You will also be asked to complete two virtual follow-up visits 12 and 16 weeks after receiving the study medication.

You will be randomized to receive one of three study medications: 400 mg/day of CBD that includes trace amounts of THC (<0.3%), 400 mg/day of CBD that includes no THC, or 400 mg/day of Hemp Seed Oil, which does not contain any CBD or THC. You will be asked to take four softgels in the morning and four softgels in the evening for a total of eight softgels per day for 8 weeks. The treatment you get will be chosen by chance, like flipping a coin. You will have an equal chance of being given each treatment. Neither you nor the study doctor will know which treatment you are getting. You should take the softgels with food. The softgels should be stored at room temperature, protected from light, in a safe place away from other people, especially children. Ingredients in the softgels you could be taking include hemp seed oil, hemp extract, gelatin, and glycerin. The softgels are not vegan because they contain a bovine (beef) gelatin.

Baseline: Clinical Translational Research Center (CTRC)

When you were scheduled for this visit, you were asked to refrain from using alcohol for 24 hours, and caffeine and nicotine for 1 hour prior to the Baseline.

During this visit, you will meet with a research assistant who will explain the research study and all the procedures required of you, as well as answer any questions that you may have. If you agree to participate in the research study, you will fill out the consent form (this form).

You will complete a breathalyzer assessment to make sure you have no alcohol in your system. If the breathalyzer reading is above 0.000, you will not be eligible to participate. You will also provide a urine sample to ensure you have not used any illicit substances and to assess THC levels in your system. If the drug screen comes back positive for substances other than cannabis you will not be eligible to participate. If you are female, the urine sample will also be used to conduct a pregnancy test. If you are pregnant or breastfeeding, you will not be eligible to participate. Individuals who engage in activities that could result in pregnancy should use an effective form of birth control until use of the study medication is complete.

A trained phlebotomist will collect approximately 24 mL (~1.6 tablespoons) of blood through an arm or hand vein, like a blood draw at your doctor's office. This blood will be used to measure cannabinoids in your blood, as well as to check your general health and liver function. If markers of your liver function (i.e., aspartate transaminase and alanine transaminase) are more than 2x the upper normal limits you may be removed from the study due to the potential for cannabinoids (e.g., THC and CBD) to affect liver function.

Your heart rate, oxygen levels, blood pressure, height, weight, waist circumference, and hip circumference will be measured by a research assistant or nurse.

A physician will conduct a physical exam to make sure that it is safe for you to start using the study medication. The research assistant will ask you questions about your medical history, medications that you are taking, as well as take you through a brief interview meant to check for psychiatric disorders and assess cannabis use disorder. You will complete surveys on an iPad that will ask questions about your demographics, general health, substance use, cannabis use disorder and withdrawal symptoms, anxiety, depression, suicidality, sleep, and expectancies about cannabis use.

The Baseline will take approximately 2 hours to complete.

After you complete the Baseline, the study physician will review the medical information (e.g., liver function blood test) collected during this visit to determine if you are eligible to continue participating in the study. If you are eligible to continue, then you will be scheduled for your first virtual therapy session.

Daily Diaries

After completing your baseline appointment, you will receive a daily email that has a link to complete a survey to record any cannabis use and withdrawal symptoms during the study. You will receive these emails until you complete the Week 8 visit, and each survey should take about five minutes to complete.

Therapy Session 1: Zoom

You will complete the first of five virtual therapy sessions about one week after the Baseline. The therapy involved in this study is based on a program developed by the U.S. Substance Abuse and Mental Health Services Administration (SAMHSA) and will focus on helping you build skills related to reducing cannabis use. All therapy sessions will be conducted by a trained therapist supervised by a licensed clinician. The initial therapy session will take approximately 1 hour to complete.

Initial Medication Dispense: CINC

You will meet with a research assistant within five days of the first virtual therapy session to receive your randomly assigned study medication and detailed instructions on using it. This visit will take approximately 30 minutes.

Weeks 1, 2, 4, & 6: CINC

One, two, four, and six weeks after receiving your study medication, you will meet with a research assistant. For each of these visits, you will be asked to not take your study medication until after the visit is complete, as well as to not use alcohol for 24 hours and not use caffeine or nicotine for 1 hour before each visit. You will complete a breathalyzer assessment to make sure you have no alcohol in your system. If the breathalyzer reading is above 0.000, you will no longer be eligible to participate. You will also be asked to bring in your medication bottle so that we can count the number of softgels you took since the last visit. The research assistant will ask you about medications that you are taking and if you have experienced any adverse events. A blood draw will also be collected to measure cannabinoids at the Week 1 visit (10mL or ~0.7 tablespoons) and to measure both cannabinoids and liver function at the Week 4 Visit (20mL or ~1.4 tablespoons). You will provide a urine sample to measure cannabinoids and to conduct a pregnancy test if you are female. You will complete surveys on an iPad that will ask questions about substance use, sleep, cannabis craving, and cannabis withdrawal symptoms. You will be given additional study medication when needed. In addition to the above procedures, during the Week 4 Visit you will complete a suicidality survey, and your heart rate, oxygen levels, blood pressure, weight, waist circumference, and hip circumference will be measured. Each of these visits will take approximately 1 hour to complete.

Virtual Therapy Sessions 2-5: Zoom

You will complete a virtual therapy session within 5 days of each of the in-person visits at CINC. Each virtual therapy session will take approximately 1 hour to complete.

Week 8: CINC

You will be asked to not take your study medication on the day of the appointment, as well as to not use alcohol for 24 hours and not use caffeine or nicotine for 1 hour before this visit. You will complete a breathalyzer assessment to make sure you have no alcohol in your system. If the breathalyzer reading is above 0.000, you will no longer be eligible to participate. You will also be asked to bring in your medication bottle so that we can count the number of softgels you took since the last visit. Any remaining study medication will be returned. A research assistant will ask you about medications that you are taking and if you have experienced any adverse events. A blood draw (20mL or ~1.4 tablespoons) will be collected to measure cannabinoids and to measure your liver function. You will provide a urine sample to measure cannabinoids and to conduct a pregnancy test if you are female. A research assistant will measure your heart rate, oxygen levels, blood pressure, height, weight, waist circumference, and hip circumference. You will complete surveys on an iPad that will ask questions about your general health, substance use, cannabis craving, cannabis use disorder and withdrawal symptoms, anxiety, depression, suicidality, and sleep.

At the end of this visit, you will be offered additional self-help resources and a range of continued treatment services available will be explained. Additionally, if you wish to pursue additional treatment, we can write you a referral, either the day of or at any future time that you desire. This final in-person study visit should last approximately 1 hour.

Weeks 12 & 16: Zoom

Twelve and sixteen weeks after receiving your study medication, you will meet with a research assistant virtually. During these meetings, the research assistant will ask you about medications that you are taking, and if you have experienced any adverse events. You will also complete surveys that will ask questions about your general health, substance use, cannabis use disorder and withdrawal symptoms, anxiety, depression, suicidality, and sleep. These meetings will take approximately 30 minutes to complete.

Voluntary Participation and Withdrawal

Whether or not you take part in this research is your choice. You can leave the research at any time, and it will not be held against you.

The person in charge of the research study can remove you from the research study without your approval. Possible reasons for removal include if the study doctor thinks that being in the study may cause you harm, if you physically or verbally abuse study staff, if you fail to comply with explicit study instructions, if you are or become pregnant, if your breath alcohol level is above 0.000 at a visit, or if you test positive for substances other than cannabis during the drug screen.

If you stop being in the research, already collected data may not be removed from the study database.

We will tell you about any new information that may affect your health, welfare, or choice to stay in the research.

Risks and Discomforts

There are some potential risks if you take part in this study:

- Unwanted side effects of hemp-derived CBD:
    - Although unlikely due to the low dose of CBD used in this study, potential side effects could include drowsiness, gastrointestinal discomfort, and changes in liver function. We will check your liver function during the study to ensure that it is in a range safe to use the study medication.
    - Although Epidiolex, which contains CBD, has been approved by the FDA for a rare seizure disorder in children, CBD is metabolized differently with expression of one metabolite that is much higher in humans than in animals. Animal safety studies have not been conducted with this metabolite and therefore the safety of this metabolite is unknown at this time.
    - Based on studies in animals, CBD has been shown to cause male reproductive organ changes that can result in reduced male fertility. Also, CBD in animals have been associated with adverse effects to the fetus and fetal development

and therefore you will not be allowed to participate in this study if you are pregnant or if you cannot use an appropriate contraception method.
  - Due to the possibility for CBD to produce sedative effects, it could impair your ability to drive or operate heavy machinery. You should not drive or operate heavy machinery after dosing if you are impaired. We will check your liver function during the study to ensure that it is in a range safe to use the study medication. The dosing procedure calls for four taking softgels at one time, two times per day. Taking all eight softgels at once could result in additional side effects.
- <u>Blood draw:</u> There is a small risk of swelling, bruising, infection, feeling light-headed, and fainting during a blood draw. These risks are minimized by having highly trained personnel perform the procedures using sterile techniques.
- <u>Confidentiality:</u> There is a risk that people outside of the research team will see your research information. We will do all that we can to protect your information, but it cannot be guaranteed. Any personal identifying information and any record linking that information to study ID numbers will be destroyed when the study is completed.
- <u>Failing a drug test:</u> One of the medications you could receive in this study contains small amounts of THC, which could show up in a drug test. If you have to complete a drug test for employment or any other reason, you should know that you could fail the drug test if they are testing for THC. Neither you nor the study team will know which medication you are using during the study.
- <u>Psychological risks completing questionnaires:</u> You may experience fatigue or emotional discomfort while completing the self-report questionnaires, some of which include sensitive questions about medical history, substance use, anxiety, depression, and suicidality. You are welcome to take breaks while completing the questionnaires if you are feeling fatigued. In addition, your participation in the research is completely voluntary, including providing answers to individual questions.

In addition to these risks, this research may hurt you in ways that are unknown. These may be minor inconveniences or may be so severe as to cause death.

It is important that you tell the Principal Investigator, Dr. Cinnamon Bidwell, if you think you have been injured because of participating in this study. ***You can call her at 303-492-0288.***

Significant New Findings

You will be informed of any significant new findings that may affect your willingness to contribute to participate in this research.

Potential Benefits

We cannot promise any benefits to you or others from your taking part in this research. However, possible benefits include reducing your cannabis use, withdrawal, and CUD symptoms.

Payment for Research Related Injury

If you need medical care because of taking part in this research study, seek medical attention immediately (if it is a medical emergency, first call 911). Generally, this care will be billed to you, your insurance, or other third party. University of Colorado Boulder has no program to pay for medical care for research-related injury. Please contact the investigator as soon as possible to report the event.

Alternatives

There may be other ways of treating CUD symptoms, including various types of therapy not included in the current study. You could also choose to get no treatment at all as well as to not participate in this study.

You should talk to your doctor about your choices. Make sure you understand all your choices before you decide to take part in this study. You may leave this study and still have these other choices available to you.

This research is not designed to formally diagnose or treat CUD. An alternative to participating in the study is to not participate in this study.

Confidentiality

Information obtained about you for this study will be kept confidential to the extent allowed by law. Research information that identifies you may be shared with the University of Colorado Boulder Institutional Review Board (IRB) and others who are responsible for ensuring compliance with laws and regulations related to research, including people on behalf of the Office for Human Research Protections.

- The Food and Drug Administration (FDA)
- The National Institute on Drug Abuse, who funds this research
- University of Colorado Anschutz, where the study doctor is based

The sponsor, monitors, auditors, the IRB, and the US Food and Drug Administration will be granted direct access to your research information to conduct and oversee the research. By signing this document, you are authorizing this access. We may publish the results of this research. However, we will keep your name and other identifying information confidential.

A description of this clinical trial will be available on http://www.ClinicalTrials.gov, as required by U.S. Law. This Web site will not include information that can identify you. At most, the Web site will include a summary of the results. You can search this Web site at any time.

This study has been issued a Certificate of Confidentiality from the federal government to help protect your privacy. This certification means that the researchers cannot be forced to

tell people who are not connected with the study, such as the court system, about your participation in this study. But, if you request that we do so, we will release information that is unique to you.

There are three exceptions to this promise of confidentiality:

1. If we see or are told information that makes us reasonably suspect that a child or at-risk adult is being or has been abused, mistreated, or neglected, we will immediately report that information to the county department of social services or a local law enforcement agency.
2. If we learn of a serious threat of imminent physical violence against a person, we will report that information to the appropriate legal authorities and make reasonable and timely efforts to notify the potential victim.
3. This promise of confidentiality does not include information we may learn about future criminal conduct.

The biological samples collected to check your general health and liver function will be labeled with your name and date of birth and sent to Boulder Community Health for analysis. All your other data and biological samples will be labeled with a unique identification number and not your name or any other information that would identify you. Instead, we will use an ID number to keep track of your data. After the study is completed, we will deidentify the data and biospecimens by removing the identifiers that link it to you. The deidentified data and biospecimens may be used for future research purposes by the Principal Investigator of this study. The deidentified data and biospecimens may also be shared with other investigators for future research.

The results of the clinical blood tests (complete blood count and comprehensive metabolic panel) will be made available to you upon request. Any clinically relevant research results, including individual research results, will be disclosed to you. Specifically, if your liver function test is outside of the range safe to use the study medication, we will share this information with you and give you a copy of your test results to share with your primary care physician.

Cost of Participation

There are no costs associated with participation in this study.

Payment for Participation

If you agree to take part in this research study, we will pay you up to $390 for your time and effort. You will receive $80 in cash for completing the Baseline visit, $40 in cash for completing the Week 1 visit, $40 in cash for the Week 2 visit, $60 in cash for the Week 4 visit, $40 in cash for the Week 6 visit, $60 in cash for Week 8 visit, and a $20 gift card for completing each of the follow-up visits ($40 gift card total across both). You will also be paid $15 if you complete at least 80% (23 of 28) of the daily diaries between the Week 1 and Week 4 visits, and $15 if you complete at least 80% (23 of 28) of the daily diaries between the Week 4 and Week 8 visits. Payments for in-person visits will be received at the end of each visit, payments for completing the daily diaries will be made at the end of the Week 4 and 8 visits, and payments for the follow-up visits will be emailed to you. If you leave the

study early, or if we must take you out of the study early, you will only be paid for visits you have completed.

Contact for Future Studies

We would like to keep your contact information on file so we can notify you if we have future research studies that we think you may be interested in. This information will be used by only the principal investigator of this study and only for this purpose.

Please initial your choice below:

___ Yes, you may contact me for future research studies. The best way to contact me is: (enter preferred telephone number and/or email address)

____

___ No, you may not contact me for future research studies.

Questions

If you have questions, concerns, or complaints, or think the research has hurt you, talk to the research team at 303-492-0288.

This research has been reviewed and approved by an IRB. You may talk to them at (303) 735-3702 or irbadmin@colorado.edu if:

- Your questions, concerns, or complaints are not being answered by the research team.
- You cannot reach the research team.
- You want to talk to someone besides the research team.
- You have questions about your rights as a research subject.
- You want to get information or provide input about this research.

Signatures

Your signature documents your permission to take part in this research.

____

Signature of subject Date

____

Printed name of subject

____

Signature of person obtaining consent Date

____

Printed name of person obtaining consent
