# Supplementary material for: LOTUS: Protocol for a double-blind placebo controlled randomized trial of hemp-derived cannabidiol for the treatment of cannabis use disorder
Source: PLoS One. 2024 Sep 30;19(9):e0308262. doi: 10.1371/journal.pone.0308262 (PMC11441658; doi:10.1371/journal.pone.0308262)
Supplement: S4 File — (PDF) [file pone.0308262.s006.pdf]

MEETING ROSTER
Interventions to Prevent and Treat Addictions Study Section
Risk, Prevention and Health Behavior Integrated Review Group
CENTER FOR SCIENTIFIC REVIEW
IPTA
06/22/2023 - 06/23/2023

Notice of NIH Policy to All Applicants: Meeting rosters are provided for information purposes only. Applicant investigators and institutional officials must not communicate directly with study section members about an application before or after the review. Failure to observe this policy will create a serious breach of integrity in the peer review process, and may lead to actions outlined in NOT-OD-22-044, including removal of the application from immediate review.

CHAIRPERSON(S)

PIPER, MEGAN E, PHD
PROFESSOR
DEPARTMENT OF MEDICINE
UNIVERSITY OF WISCONSIN-MADISON
MADISON, WI 53711

MEMBERS

ALETRARIS, LYDIA, PHD *
ASSOCIATE RESEARCH SCIENTIST
SCHOOL OF SOCIAL WORK
UNIVERSITY OF GEORGIA
ATHENS, GA 30602

BERNSTEIN, STEVEN L, MD
PROFESSOR
DEPARTMENT OF EMERGENCY MEDICINE
GEISEL SCHOOL OF MEDICINE AT DARTMOUTH
LEBANON, NH 03756

BINSWANGER, INGRID A, MD
SENIOR CLINICIAN INVESTIGATOR
INSTITUTE FOR HEALTH RESEARCH
KAISER PERMANENTE
AURORA, CO 80014

BRICKER, JONATHAN B, PHD
PROFESSOR
DIVISION OF PUBLIC HEALTH SCIENCES
FRED HUTCHINSON CANCER RESEARCH CENTER
UNIVERSITY OF WASHINGTON
SEATTLE, WA 98109

CLAUS, ERIC D, PHD
ASSOCIATE PROFESSOR
DEPARTMENT OF BIOBEHAVIORAL HEALTH
THE PENNSYLVANIA STATE UNIVERSITY
UNIVERSITY PARK, PA 16802

FENDRICH, MICHAEL, PHD *
SCIENTIFIC DIRECTOR
ADVOCATE AURORA RESEARCH INSTITUTE
MILWAUKEE, WI 53211

GAINES, TOMMI LYNN, DRPH *
ASSOCIATE PROFESSOR
DIVISION OF GLOBAL PUBLIC HEALTH
DEPARTMENT OF MEDICINE
SCHOOL OF MEDICINE
UNIVERSITY OF CALIFORNIA, SAN DIEGO
LA JOLLA, CA 92093

GARRISON, KATHLEEN A, PHD
ASSISTANT PROFESSOR
DEPARTMENT OF PSYCHIATRY
YALE SCHOOL OF MEDICINE
NEW HAVEN, CT 06510

GRYCZYNSKI, JAN, PHD
SENIOR RESEARCH SCIENTIST
FRIENDS RESEARCH INSTITUTE
BALTIMORE, MD 21201

HAGLE, HOLLY *
ASSOCIATE RESEARCH PROFESSOR
MISSOURI CENTER FOR ADDICTION RESEARCH
AND ENGAGEMENT
COLLEGE OF ARTS AND SCIENCE
UNIVERSITY OF MISSOURI
KANSAS CITY, MO 64110

JAGER, JUSTIN O, PHD *
ASSOCIATE PROFESSOR
COLLEGE OF HEALTH SOLUTIONS
THE SANFORD SCHOOL
ARIZONA STATE UNIVERSITY
TEMPE, AZ 85281

KELLEY, MICHELLE L, PHD *
PROFESSOR AND CHAIR
DEPARTMENT OF PSYCHOLOGY
COLLEGE OF SCIENCES
OLD DOMINION UNIVERSITY
NORFOLK, VA 23322

KOWALCHUK, ALICIA ANN, DO *
ASSOCIATE PROFESSOR
BAYLOR COLLEGE OF MEDICINE
HOUSTON, TX 77030

LANGDON, KIRSTEN JOHNSON, PHD *
ASSISTANT PROFESSOR
DEPARTMENT OF BEHAVIORAL AND SOCIAL SCIENCES
DEPARTMENT OF PSYCHIATRY AND HUMAN BEHAVIOR
BROWN UNIVERSITY
PROVIDENCE, RI 02912

LEDGERWOOD, DAVID M, PHD
PROFESSOR
DEPARTMENT OF PSYCHIATRY
AND BEHAVIORAL NEUROSCIENCES
SCHOOL OF MEDICINE
WAYNE STATE UNIVERSITY
DETROIT, MI 48201

Notice of NIH Policy to All Applicants: Meeting rosters are provided for information purposes only. Applicant investigators and institutional officials must not communicate directly with study section members about an application before or after the review. Failure to observe this policy will create a serious breach of integrity in the peer review process, and may lead to actions outlined in NOT-OD-22-044, including removal of the application from immediate review.

MATHEW, AMANDA R, PHD *
ASSISTANT PROFESSOR
DEPARTMENT OF PREVENTIVE MEDICINE
RUSH UNIVERSITY
CHICAGO, IL 60612

MCHUGH, REBECCA KATHRYN, PHD
ASSOCIATE PROFESSOR
DEPARTMENT OF PSYCHIATRY
HARVARD MEDICAL SCHOOL
BELMONT, MA 02478

MCNEELY, JENNIFER, MD
ASSOCIATE PROFESSOR
DEPARTMENTS OF POPULATION HEALTH AND MEDICINE
GROSSMAN SCHOOL OF MEDICINE
NEW YORK UNIVERSITY
NEW YORK, NY 10016

MCPHERSON, STERLING M, PHD
DIRECTOR AND PROFESSOR
PROGRAM OF EXCELLENCE IN ADDICTIONS RESEARCH
DEPARTMENT OF COMMUNITY AND BEHAVIORAL HEALTH
ELSON S. FLOYD COLLEGE OF MEDICINE
WASHINGTON STATE UNIVERSITY
SPOKANE, WA 99210

MILLS, SARAH, PHD, MPH *
ASSISTANT PROFESSOR
DEPARTMENT OF HEALTH BEHAVIOR
GILLINGS SCHOOL OF GLOBAL PUBLIC HEALTH
UNIVERSITY OF NORTH CAROLINA
CHAPEL HILL, NC 27599

MONTGOMERY, LATRICE, PHD
DIRECTOR OF CLINICAL RESEARCH AT RIA HEALTH,
ADJUNCT ASSOCIATE PROFESSOR
DEPARTMENT OF PSYCHIATRY AND
BEHAVIORAL NEUROSCIENCE
COLLEGE OF MEDICINE
UNIVERSITY OF CINCINNATI
CINCINNATI, OH 45229

MUMBA, MERCY N, PHD
ASSOCIATE PROFESSOR
CAPSTONE COLLEGE OF NURSING
THE UNIVERSITY OF ALABAMA
TUSCALOOSA, AL 35401

NAPPER, LUCY ELIZABETH, PHD *
ASSOCIATE PROFESSOR
DEPARTMENT OF PSYCHOLOGY
COLLEGE OF ARTS AND SCIENCES
LEHIGH UNIVERSITY
BETHLEHEM, PA 18015

POWELL, TERRINIEKA WILLIAMS, PHD *
ASSOCIATE PROFESSOR
POPULATION, FAMILY AND REPRODUCTIVE HEALTH
BLOOMBERG SCHOOL OF PUBLIC HEALTH
JOHNS HOPKINS UNIVERSITY
BALTIMORE, MD 21205

RAIFF, BETHANY R, PHD *
PROFESSOR
DEPARTMENT OF PSYCHOLOGY
COLLEGE OF SCIENCE AND MATHEMATICS
ROWAN UNIVERSITY
GLASSBORO, NJ 08028

SADASIVAM, RAJANI, PHD
PROFESSOR
DIVISION OF HEALTH AND IMPLEMENTATION SCIENCE
DEPARTMENT OF POPULATION AND QUANTITATIVE
HEALTH SCIENCES
UNIVERSITY OF MASSACHUSETTS MEDICAL SCHOOL
WORCESTER, MA 01605

SARAIYA, TANYA CHANDRESH, PHD *
ASSISTANT PROFESSOR
DEPARTMENT OF ALCOHOL & SUBSTANCE USE STUDIES
GRADUATE SCHOOL OF APPLIED AND PROFESSIONAL
PSYCHOLOGY
RUTGERS UNIVERSITY
PISCATAWAY, NJ 08854

SHEFFER, CHRISTINE ELIZABETH, PHD
PROFESSOR
DEPARTMENT OF HEALTH BEHAVIOR
ROSWELL PARK COMPREHENSIVE CANCER CENTER
BUFFALO, NY 14263

STATON, MICHELE, PHD
PROFESSOR
DEPARTMENT OF BEHAVIORAL SCIENCES
COLLEGE OF MEDICINE
UNIVERSITY OF KENTUCKY
LEXINGTON, KY 40536

MEETING ROSTER
Interventions to Prevent and Treat Addictions Study Section
Risk, Prevention and Health Behavior Integrated Review Group
CENTER FOR SCIENTIFIC REVIEW
IPTA
06/22/2023 - 06/23/2023

Notice of NIH Policy to All Applicants: Meeting rosters are provided for information purposes only. Applicant investigators and institutional officials must not communicate directly with study section members about an application before or after the review. Failure to observe this policy will create a serious breach of integrity in the peer review process, and may lead to actions outlined in NOT-OD-22-044, including removal of the application from immediate review.

STORMSHAK, ELIZABETH A, PHD
PROFESSOR
COLLEGE OF EDUCATION
PREVENTION SCIENCE INSTITUTE
UNIVERSITY OF OREGON
EUGENE, OR 97403

VILARDAGA, ROGER, PHD
ASSOCIATE PROFESSOR
DEPARTMENT OF PSYCHIATRY AND BEHAVIORAL SCIENCES
SCHOOL OF MEDICINE
DUKE UNIVERSITY
DURHAM, NC 27710

WATKINS, KATHERINE E, MSHS, MD *
SENIOR SCIENTIST
HEALTH CARE DIVISION
RAND CORPORATION
SANTA MONICA, CA 90407

WELSH, JUSTINE WITTENAUER, MD *
DIRECTOR OF THE EMORY HEALTHCARE ADDICTION SERVICES
DEPARTMENT OF PSYCHIATRY AND BEHAVIORAL SCIENCES
SCHOOL OF MEDICINE
EMORY UNIVERSITY
ATLANTA, GA 30307

YI, RICHARD, PHD
PROFESSOR AND DIRECTOR
COFRIN LOGAN CENTER FOR ADDICTION RESEARCH AND TREATMENT
DEPARTMENT OF PSYCHOLOGY
UNIVERSITY OF KANSAS
LAWRENCE, KS 66045

MAIL REVIEWER(S)

D'SOUZA, DEEPAK CYRIL, MD
PROFESSOR
DEPARTMENT OF PSYCHIATRY
YALE UNIVERSITY
WEST HAVEN, CT 06516

SCIENTIFIC REVIEW OFFICER

VIDAL, SARAH, PHD
SCIENTIFIC REVIEW OFFICER
CENTER FOR SCIENTIFIC REVIEW
NATIONAL INSTITUTES OF HEALTH
BETHESDA, MD 20892

EXTRAMURAL SUPPORT ASSISTANT

AMARE, MERON ERMIAS
LEAD EXTRAMURAL SUPPORT ASSISTANT
CENTER FOR SCIENTIFIC REVIEW
NATIONAL INSTITUTES OF HEALTH
BETHESDA, MD 20892

* Temporary Member. For grant applications, temporary members may participate in the entire meeting or may review only selected applications as needed.

Consultants are required to absent themselves from the room during the review of any application if their presence would constitute or appear to constitute a conflict of interest.
