# Supplementary material for: LOTUS: Protocol for a double-blind placebo controlled randomized trial of hemp-derived cannabidiol for the treatment of cannabis use disorder
Source: PLoS One. 2024 Sep 30;19(9):e0308262. doi: 10.1371/journal.pone.0308262 (PMC11441658; doi:10.1371/journal.pone.0308262)
Supplement: S5 File — (PDF) [file pone.0308262.s007.pdf]

July 7, 2024

Dear Editorial Board,

My co-authors and I are pleased to submit our manuscript “LOTUS: Protocol for a double-blind placebo controlled randomized trial of hemp-derived cannabidiol for the treatment of cannabis use disorder” for consideration as an original clinical trial protocol submission in *PLOS ONE*. As cannabis legalization continues to spread across the United States, average Δ^9-tetrahydrocannabinol concentrations in recreational products have significantly increased, and no prior study has evaluated effective treatments to reduce cannabis use among high potency cannabis users. Some research has found that the non-intoxicating cannabinoid cannabidiol reduces cannabis use and cannabis use disorder-related symptoms, such as affective disturbance and withdrawal. Results of these studies are promising but limited to synthetic or isolated forms of cannabidiol. Thus, this study will make a significant contribution to the literature through the conduct of a placebo-controlled randomized control trial comparing the effects of hemp-derived cannabidiol on reducing Δ^9-tetrahydrocannabinol use in concentrate users with cannabis use disorder.

Our group has been working productively in this area of research for quite some time. Most recently, we published the results of the first randomized study to examine the effects of *ad libitum* legal market cannabis use on anxiety symptoms (Bidwell et al, 2024). Results of that study demonstrated that a*d libitum* use of CBD-dominant cannabis was associated with greater short- and long-term anxiolytic effects as compared to THC+CBD and THC-dominant cannabis, both immediately after use and over a 4-week exposure period. Our current and prior research adds sorely needed, fully powered trial data to the field, and the LOTUS protocol stands to extend this work towards improving therapeutic approaches to treating individuals with problematic high potency cannabis use.

This is the first interaction with *PLOS* regarding the submitted manuscript. This manuscript has not and will not be submitted for publication until a decision is made regarding its acceptability for publication in *PLOS ONE*. Potential editors who may be suitable for review of our protocol manuscript could include Dr. Lion Shahab, Dr. Dolly Baliunas, or Dr. Benjamin Bearnot. This study is primarily funded by NIDA award R01DA059234 (MPI: Bidwell & Hutchison) and all requested funding information (e.g., funding award letter, list of NIDA reviewers) has been included with the submission in addition to copies of the complete IRB-approved protocol and consent form. The protocol has been pre-registered on ClinicalTrials.gov (NCT06107062).

No other papers have been published for this study. If accepted for publication, it will not be published elsewhere. The work in its entirety is in compliance with ethical standards governing the treatment of human research participants. All authors have made a significant contribution to the manuscript. All authors have also reviewed this most current version and approve of its content. There are no conflicts of interest. I will be the corresponding author for this submission. We look forward to hearing from you regarding the editorial decision. Thank you very much.

Sincerely,

L. Cinnamon Bidwell, Ph.D.
Assistant Professor
Institute of Cognitive Science
University of Colorado at Boulder

Bidwell LC, Martin-Willett R, Skrzynski C, Lisano J, Ortiz Torres M, Giordano G, Hutchison KE & Bryan AD. (2024). Acute and extended anxiolytic effects of CBD in cannabis flower: A quasi-experimental ad libitum use study. *Cannabis and Cannabinoid Research*. PMID: 38252547.

Department of Psychology & Neuroscience and Institute of Cognitive Science
594 UCB • Boulder, Colorado 80309-0000
t 303 735 5180 • renee.martinwillett@colorado.edu
