# Supplementary material for: LOTUS: Protocol for a double-blind placebo controlled randomized trial of hemp-derived cannabidiol for the treatment of cannabis use disorder
Source: PLoS One. 2024 Sep 30;19(9):e0308262. doi: 10.1371/journal.pone.0308262 (PMC11441658; doi:10.1371/journal.pone.0308262)
Supplement: S1 Protocol — (PDF) [file pone.0308262.s008.pdf]

TITLE: Hemp-derived cannabidiol for the treatment of cannabis use disorder: A double-blind placebo-controlled randomized trial

PROTOCOL VERSION DATE: March 25, 2024
VERSION: 2

PRINCIPAL INVESTIGATOR (PI):

Name: L. Cinnamon Bidwell, PhD
Telephone: 303-735-5180
Email**: lcb@colorado.edu**

KEY PERSONNEL

Name: Angela Bryan, PhD
Role in project: Co-investigator

Name: Kent Hutchison, PhD
Role in project: Co-investigator

Name: Rachael Rzasa-Lynn, MD, University of Colorado Anschutz
Role in project: Co-Investigator, Study Physician

Name: Jonathan Lisano, PhD
Role in project: Research Coordinator

Name: Gregory Giordano, MS
Role in project: Research Coordinator

Name: Renée Martin-Willett, MA
Role in project: Research Coordinator, Study Therapist

Name: Samantha Natal
Role in project: Research Coordinator, Study Therapist

Name: Kyle Chrystal
Role in project: Research Coordinator

Name: Elicia Azua
Role in project: Research Assistant

Name: Paige Phillips
Role in project: Research Assistant

Name: Austin Drake
Role in project: Research Assistant

Name: Devon Rapken

Role in project: Research Assistant

Name: Evangelique Lopez
Role in project: Research Assistant

Name: Gray MacDonald
Role in project: Research Assistant

Name: Shelly Dey
Role in project: Research Assistant

Name: Nelly Mulenga
Role in project: Research Assistant

Name: John Whitten
Role in project: Research Assistant

GENERAL RESEARCH STAFF

1-4 undergraduate research assistants will assist with this protocol at a time. The PI will ensure that appropriate CITI and protocol training is maintained. Responsibilities of the undergraduate research assistants will include assisting in recruitment efforts by distributing recruitment materials, screening and scheduling potential participants, entering and double-checking data, and conducting surveys.

I. OBJECTIVES

As cannabis legalization continues to spread across the United States, average Δ^9-tetrahydrocannabinol (THC) concentrations in recreational products have significantly increased, with THC levels as high as 90-95%.[1,2] Our preliminary data suggest that concentrate use elicits blood THC levels more than twice as high as cannabis flower use, and that concentrate use is associated with greater withdrawal, tolerance, and Cannabis Use Disorder (CUD),[3] prompting concern about the risks of these high potency products in relation to problem use and CUD. No prior study has evaluated effective treatments to reduce cannabis use in this high risk group.

Several previous studies have found that the non-intoxicating cannabinoid cannabidiol (CBD), which may antagonize the effects of THC on CB_1 and CB_2 receptors, reduces cannabis use and CUD-related symptoms, such as affective disturbance and withdrawal.[4,5] Results of these studies are promising, but limited to synthetic or isolated forms of CBD that are not widely available. There have been no tests of the hemp-derived CBD that is widely available without a prescription across the U.S. Importantly, hemp-derived CBD comes in two forms, one with a small amount of THC (~0.3% THC, full spectrum; fsCBD) and one without THC (0% THC; broad spectrum; bsCBD). It is possible that a small amount of THC may confer additional benefits with respect to withdrawal and related affective disturbance, and in turn be beneficial for reducing THC use overall.[4] Consistent with this hypothesis, pilot data from our lab suggest that CBD, that also contains low levels of THC, reduces THC drug reward, withdrawal, anxiety, and overall THC use in heavy concentrate users,[6] supporting the potential for hemp-derived CBD to reduce

THC use and mitigate withdrawal in this high risk group. However, no placebo-controlled trial has been conducted comparing hemp-derived CBD with and without THC on reducing THC use.

The overarching goal of this study is to conduct a placebo-controlled RCT comparing the effects of hemp-derived CBD (fsCBD vs. bsCBD vs. placebo) on reducing THC use in concentrate users with CUD. Specifically, we have the following aims:

Aim 1. Test the effect of bsCBD (400 mg), fsCBD (400 mg), and placebo, on THC use and CUD symptoms over eight weeks of use.

Hypothesis 1a. Both fsCBD and bsCBD, relative to placebo, will significantly reduce total mg of THC used and urine THC-COOH levels at 1, 2, 4, 6, and 8 weeks and CUD symptoms at 8 weeks after baseline.

Hypothesis 1b. fsCBD, relative to bsCBD, will significantly reduce total mg of THC used and urine THC-COOH levels at 1, 2, 4, 6, and 8 weeks and CUD symptoms at 8 weeks after baseline.

Aim 2. Test the effect of fsCBD and bsCBD, relative to placebo, on mechanisms that may underlie their effects on reducing cannabis use, specifically affective, physiological, and physical withdrawal symptoms over the eight week trial.

Hypothesis 2a. Both fsCBD and bsCBD, relative to placebo, will significantly reduce all three facets of withdrawal symptoms at 1, 2 and 4 weeks after baseline.

Hypothesis 2b. fsCBD, relative to bsCBD, will significantly reduce the three facets of withdrawal symptoms at 1, 2 and 4 weeks after baseline.

Exploratory Aim 3. To test whether the effects of fsCBD and bsCBD on reducing THC use over 8 weeks are mediated through their effects on reducing affective, physiological, and physical facets of withdrawal from week 1 to week 4.

II. BACKGROUND AND SIGNIFICANCE

Use of high-potency cannabis concentrates is rapidly increasing. The *Cannabis sativa* L. plant contains hundreds of phytocannabinoids, but arguably of greatest importance to public health risk is the psychoactive cannabinoid Δ^9-tetrahydrocannabinol (THC). THC is associated with numerous risks, including for cannabis use disorder (CUD), cognitive harm, affective disturbance, and psychotomimetic symptoms.[7] In the wake of recreational cannabis legalization across the U.S., there has been an increase in the availability and use of concentrated cannabis products (or “concentrates”), that are made by extracting cannabinoids from the plant into resins and waxes with THC concentrations as high as 90-95%[1,2]. Point-of-sale data from recreational and medical dispensaries in Colorado (Figure 1) indicate that in the first five years that legal markets were open, concentrate sales increased 409% (from $113 million to $575 million in annual sales), while flower and edible sales have increased 80% and 234% respectively during the same period. That is, concentrates made up just 17% of the market in 2014 and five years later made up 35% of the cannabis market in

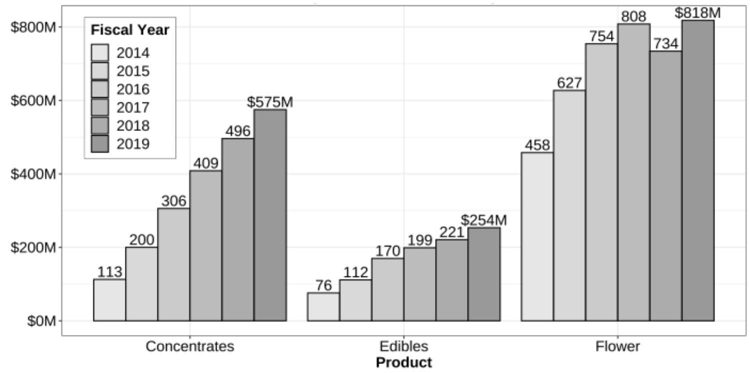

Figure 1. Sales of cannabis products by product type in Colorado between 2014 and 2019. Source: CO Department of Revenue.

Colorado. However, although concentrate use is popular among cannabis users, particularly in states with legal recreational markets, very little research has been done to examine their effects.

Cannabis concentrate use leads to heavy THC exposure and greater harm. Observational research indicates that concentrate use is associated with significantly greater harms than flower use, likely due to its greater THC content. Our and other groups' data suggest that use of cannabis with greater THC concentrations may increase the harms associated with cannabis use and contribute to greater dependence, withdrawal, and affective disturbance in concentrate users, over and above frequency of cannabis use more broadly.[8–17] Notably, these risks exist on the extreme end of a broader continuum of well-documented harms from cannabis use. Specifically, more frequent use of cannabis with higher THC content is associated with more severe CUD symptoms, and higher doses of THC are also more likely to produce anxiety, agitation, paranoia, and psychosis.[13] Among adolescents, concentrate use predicted a greater likelihood of persistent and more frequent cannabis use at one-year follow-up than use of any other cannabis product.[17] Finally, our work has indicated that frequent concentrate users, relative to frequent flower users, report greater withdrawal, more total CUD symptoms, and are specifically more likely to endorse the Diagnostic and Statistical Manual of Mental Disorders, 5th edition (DSM-5) tolerance and loss of control diagnostic criteria.[1] No previous work has explicitly evaluated effective treatments for reducing cannabis use and THC exposure in this group of high-risk users, who are most likely to benefit from an effective harm-reduction intervention.

Cannabidiol as a candidate medication for CUD. THC is the putatively harmful component of cannabis and its agonism of the CB_1 receptor is thought to underlie many of the intoxicating, impairing, and negative affect-inducing actions of cannabis.[18,19] Accordingly, a non-intoxicating compound that alters the effects of THC or endocannabinoids at CB_1 receptors could be useful for mitigating harms and reducing cannabis use.[4,5] One promising candidate CUD medication is the phytocannabinoid cannabidiol (CBD), which is anxiolytic and may regulate the reinforcing and motivational aspects of cannabis and other drugs.[20] CBD can be extracted from the cannabis plant (often from the hemp cultivar, which naturally contains lower THC concentrations) or pharmaceutically synthesized. Critically, unlike THC, human laboratory studies have consistently demonstrated that CBD has no intoxicating effects[21,22] and little abuse liability among cannabis users.[23]

The pharmacological basis of CBD's potential therapeutic effects on substance use and addictive behavior is a topic of ongoing research. Unlike THC, which is a CB_1 and CB_2 partial agonist, CBD is a high-potency CB_1 and CB_2 inverse agonist that antagonizes the effects of full CB_1 and CB_2 agonists. CB_1 receptors are densely expressed in brain areas associated with drug reward and reward memory, including the nucleus accumbens (NAcc), substantia nigra, and hippocampus,[24] and inverse agonism of these receptors may disrupt drug reward processes. The CB_1 antagonist rimonabant was previously thought to hold promise for CUD treatment,[25] but development of this compound was halted after serious adverse effects emerged. In contrast, CBD may represent a better tolerated compound that achieves similar effects on CB_1 signaling and expression. CBD also increases availability of the endocannabinoid anandamide (AEA), potentially through inhibition of the AEA-hydrolyzing enzyme FAAH,[26] and acts at a variety of other potentially relevant molecular targets, including the orphan G-protein-coupled receptor GPR55 and the 5-HT_{1A} receptor.[27]

Withdrawal: A mechanism of action for CBD to reduce THC use? The DSM5 now recognizes cannabis withdrawal[13,17] as its own diagnosis, given when an individual reports at least two psychological symptoms (e.g., irritability, anxiety, depressed mood, changes in eating or sleeping) and one physiological symptom (e.g., sweatiness, shakiness, chills, headache) after stopping heavy and prolonged cannabis use. Overall, pre-clinical and human work indicate that THC is the pharmacological constituent of cannabis underlying the phenomenon of cannabis

withdrawal and related negative affect. Cessation of chronically administered THC elicits withdrawal, and withdrawal is suppressed in a dose-dependent fashion when THC is re-administered. Thus, higher THC exposure in concentrate users is linked with greater levels of withdrawal. As a result of more severe withdrawal, concentrate users may be more motivated to resume cannabis use following abstinence to suppress withdrawal symptoms, which could ultimately lead to more regular, heavy, and persistent patterns of use. Given the potential clinical significance of withdrawal and affective symptoms in the development of CUD, finding a treatment that addresses withdrawal is critical. Due to its strong anxiolytic[28] effects and its ability to modulate cannabinoid signaling in drug reward areas,[29,30] CBD has tremendous potential to reduce cannabis withdrawal and reuptake. In a preclinical model of opioid dependence, a single 5 mg/kg dose of CBD inhibited heroin-seeking behavior during reinstatement.[29] Remarkably, this effect persisted for two weeks after CBD administration. When translated to opioid-dependent humans, a single oral CBD dose (400 or 800 mg) reduced craving and anxiety, and three days of CBD treatment at these doses resulted in persistent effects a week later.[30]

While a small emerging literature exists on this question, studies summarized in Table 1 below support the potential for CBD and low dose THC to mitigate cannabis withdrawal. Further, a handful of previous studies evaluating synthetic THC[31] provide further signal for low dose THC to improve withdrawal in cannabis users. Thus, CBD combined with low dose THC might reduce withdrawal symptoms in abstaining cannabis users and this mechanism of action may drive the therapeutic effects of hemp-derived CBD on reducing cannabis use. This mechanism for reducing THC use may be particularly important in a population of high potency cannabis concentrate users who report greater withdrawal, affective disturbance, and CUD.

Table 1. Previous studies of CBD effects on THC/cannabis use and withdrawal.

Author, yr	Formulation/dose	Population	Outcome(s)	Results
Significant Treatment Effects/Positive studies				
Trigo, 2016[32]	Nabiximols (oral spray, fixed dose vs. self-titrated to a max of 108 mg THC/100 mg CBD) x 5 days	Non-treatment-seeking	Cannabis withdrawal, craving	Fixed dose Nabiximols, relative to placebo, ↓ withdrawal/craving over 5 days. Self-titrated Nabiximols, relative to placebo, prevented increase in withdrawal over 5 days
Allsop, 2014[33]	Nabiximols (oral spray, max dose 86.4 mg THC/80 mg CBD) x 6 days	Treatment-seeking CUD	Craving, withdrawal, cannabis use	Nabiximols, relative to placebo, ↓ withdrawal, craving over 6 days. No effect on cannabis use at 28-day follow-up.
Linterzis, 2019[34]	Nabiximols (oral spray, max dose 86.4 mg THC/80 mg CBD) x 12 weeks	Treatment-seeking CUD	Cannabis use, craving	Nabiximols, relative to placebo, ↓ cannabis use days over 12 weeks. No effect on craving.
Freeman, 2020[35]	Synthetic CBD (200/400/800 mg oral) x 4 weeks	Treatment-seeking CUD	Cannabis use	400 and 800 mg CBD, relative to placebo, ↓ THC- COOH, ↑ abstinence (no effect of 200 mg).
Null Treatment Effects/Negative Studies				
Haney, 2016[36]	Synthetic CBD (200/400/800 mg oral) + 5% THC x 1 dose	Non-treatment-seeking	Subjective effects, THC self-admin	No effect of any CBD dose on THC subjective effects (euphoria, HR) or THC self-administration.
Solowij, 2018[37]	Plant-derived CBD (200 mg oral) x 10 weeks (open-label)	Non-treatment-seeking	Subjective effects, cannabis use	Relative to baseline, end-of-treatment cannabis-induced euphoria and psychotic-like symptoms ↓. No effect on

				cannabis use quantity or frequency.
Trigo, 2018[38]	Nabiximols (oral spray, max dose 113.4 mg THC/105 mg CBD) x 12 weeks + MET/CBT	Treatment-seeking CUD	Cannabis use, withdrawal	No effect of nabiximols on cannabis use days, amount used, abstinence, withdrawal symptoms, THC, 11-OH-THC, THC-COOH, or CBN. Nabiximols, relative to placebo, ↓ craving.

Effects of CBD on THC use and withdrawal. The proposed study will investigate hemp-derived CBD effects among concentrate users on primary clinically relevant outcomes: reduced THC use, withdrawal, and CUD. Table 1 summarizes recent studies of CBD effects on these outcomes. The results have been mixed, with four studies reporting positive results (i.e., that CBD reduced one or more of these cannabis outcomes) and three reporting negative results. One factor that differentiates the positive and null studies is the CBD dose tested. Among the studies that tested a CBD monotherapy (no THC), effects were found at oral CBD doses of 400 mg or higher.[32,35] Specifically, a recent groundbreaking RCT from Freeman et al demonstrated a clear signal for 4 weeks of 400 mg of synthetic CBD in reducing THC use and THC-COOH in a treatment seeking CUD population.[35] Compared with placebo, a daily 400 mg dose of synthetic CBD decreased urine THC-COOH by 94.21 ng/mL and increased abstinence from cannabis by 0.48 days per week. Additionally, no effects were found at lower oral synthetic doses (200 mg) and results suggested a .99 probability that 400 mg was the most efficacious dose.

In addition, three of four positive studies tested nabiximols (Sativex), an oromucosal spray that combines plant-derived THC and CBD in a 1:1 ratio that patients can self-administer up to a predetermined maximum daily dose.[32–34] Of the two positive nabiximols studies that directly measured effects on withdrawal, both reported significant reductions in withdrawal. Randomized controlled trials of nabiximols for a variety of indications have found very low frequency (~2.2%) of intoxication, dependence, and tolerance, and together this suggests that the presence of low dose THC might reduce cannabis use at least in part via effects on mitigating withdrawal. Further, low dose THC may alter CBD pharmacokinetics and/or the dose needed to achieve therapeutic effects.[4]

Of the three null studies, one tested the effects of a range of higher oral CBD doses (200-800 mg) on THC subjective effects and self-administration, but only for a single administration session.[36] A second small open label study found effects of higher dose plant-derived CBD monotherapy (200 mg) on subjective effects of cannabis, but not long term frequency or quantity of cannabis use in non-treatment seeking users.[37] Finally, a small nabiximols study that found no difference between nabiximols (n=13) and placebo (n=12) on cannabis use or cannabinoid biomarker levels among treatment-seeking users paired both medication conditions with 12 weeks of motivational enhancement therapy with cognitive behavioral therapy (MET+CBT); this strong behavioral intervention might have obscured the pharmacological effects of cannabinoids. Notably, while not statistically significant in this small trial, the study did report a signal for CBD, with a 70.5% reduction in cannabis use in the nabiximols group vs a 42.6% reduction in cannabis use in the placebo group. [38]

Collectively, these data suggest promise for plant-derived CBD (at doses of 400 mg or higher) to reduce THC use and mitigate withdrawal among concentrate users. Further, a clear question that remains unanswered by the existing empirical literature is whether the effects of high dose CBD on THC use reduction and withdrawal are improved or worsened with a small amount of THC.

Public health significance and potential of hemp-derived CBD. Notably, while plant-derived nabiximols shows promise for reducing withdrawal and to some extent use, the study reporting the most sustained effects of CBD (400 and 800 mg) on cannabis use reduction tested high doses of a *synthetic* CBD monotherapy formulation.[35] Additionally, for many of the broad indications for which synthetic CBD monotherapy has been tested, there is a narrow therapeutic

window, such that too-low and too-high doses are ineffective.[39] In contrast, hemp-derived CBD extracts contain other minor phytocannabinoids and terpenes, including tetrahydrocannabivarin (THCV), cannabigerol (CBG) and cannabichromene (CBC), that may synergize with CBD to increase its bioavailability and efficacy and widen its therapeutic window. Thus, there are clear signals for synthetic CBD monotherapy formulations and strong hints that use of hemp-derived formulations may improve CBD's effects. Research on hemp-derived CBD has enormous public health significance, not just because the field lacks an effective CUD medication, but also because hemp-derived products with CBD and THC are already widely available to consumers. These include products referred to as full spectrum CBD (fsCBD) that have a small amount of THC (~0.3% THC) as well as other cannabinoids; and other products referred to as broad spectrum CBD (bsCBD) that are produced in a way that excludes THC, but still includes CBD and other cannabinoids. Despite their promise and wide availability, the effects of high doses of hemp-derived CBD with and without THC on reducing THC use are relatively unexplored. Given the availability of these products, even modest effects on use would have an enormous public health impact.

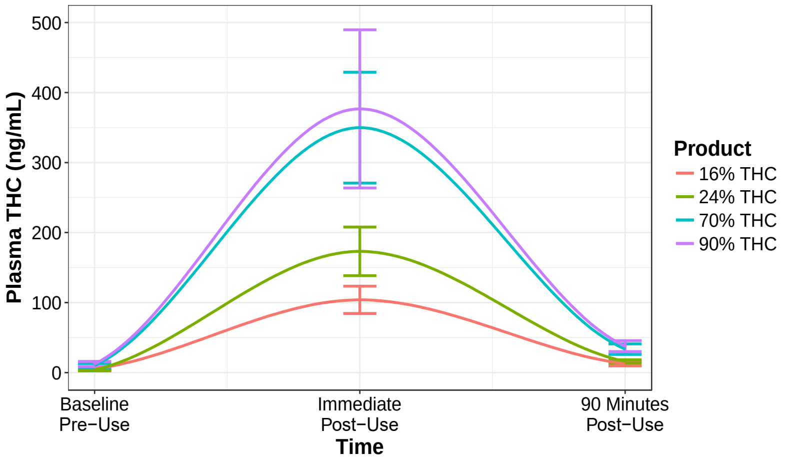

Figure 2. THC blood levels following acute use of cannabis concentrates (70-90% THC) vs. flower (16-24% THC).

Summary of significance and potential impact. Extant data indicate that cannabis concentrate use is rapidly increasing and that heavy THC exposure leads to greater cannabis-related harms, including more cannabis use, withdrawal, and CUD symptoms. While the field currently has no existing medication to treat CUD, the non-intoxicating cannabinoid CBD shows promise as a candidate CUD medication and may reduce cannabis use and withdrawal, especially if higher doses and/or hemp-derived fsCBD are used. Considering the emerging signals from clinical trials and the wide availability of bs/fsCBD on legal markets, the clear next step is to extend these findings to test 400 mg of a widely available plant-derived CBD formulation with and without THC in a rigorous RCT framework. Thus, this study will address the significant public health harms of high THC potency concentrate use and the need for a CUD medication by conducting a placebo-controlled RCT comparing the effects of hemp-derived fsCBD versus bsCBD on reducing THC use and withdrawal. We focus on concentrate users with CUD, an understudied group who are both a priority research area and the most likely to benefit from an effective cannabis harm reduction intervention.

III. PRELIMINARY STUDIES

PI Bidwell and her team have been conducting innovative cannabis research for more than 10 years and have developed cutting-edge study designs to examine the effects of cannabis products available in the legal market in Colorado. Our previous publications include studies on the acute effects of cannabis and psychiatric comorbidities, substance use RCTs and treatment trials, and neurobiological variability that influence these short and long term effects. Below, we summarize the results of recently completed as well as in-progress studies that have tested the

effects of concentrates or CBD on cannabinoid exposure, withdrawal, and other cannabis effects.

Table 2. Cannabis Use Patterns in Concentrate and Flower Users

	Concentrate n=273	Flower n=374
Cannabis Use Days (past 30 days)	22.6 (9.3)	22.7 (8.4)
Cannabis Use Disorder symptoms	3.2 (2.1)	2.9 (2.7)

Ability to recruit concentrate users with CUD. We have successfully recruited concentrate users for many federal and state-funded large scale studies on the health effects of cannabis. For example, we have recruited and retained groups of predominantly flower and concentrate users (at least four times/week of preferred form) that do not differ on overall cannabis use days per month (Table 2), establishing our ability to successfully recruit a sample of concentrate users with CUD for the current study.

Differential effects of concentrates vs. flower. We recently completed a set of studies comparing high THC legal market concentrate and flower products utilizing our mobile laboratory.[3] In our team's *JAMA Psychiatry* publication from these studies, we randomly assigned 121 individuals to naturalistic use of flower or concentrate products with different THC potencies (16%, 24%, 70%, 90%). As expected, concentrates acutely elicited blood THC levels more than twice as high as flower (mean concentrate level = 320 ng/mL; mean flower level = 140 ng/mL), with some concentrate users achieving blood levels 15-20 times greater than the mean of the flower users (Figure 2). However, THC content did not affect subjective intoxication (feeling "high") or cognitive measures such as verbal recall. These results suggest that concentrate users have much higher THC exposure and may develop higher tolerance to THC's negative effects. In addition, participants weighed their product before and after *ad libitum* use; these naturalistic administration data suggest that concentrate users use an average of 88 (SD=11) mg of THC with a single concentrate use session. Thus, there is significant room to reduce the level of THC exposure in regular concentrate users, even with use of an fsCBD product that contains low levels of THC (~0.03%).

Withdrawal in Cannabis Concentrate Users. We are conducting preliminary research on the processes underlying abstinence and withdrawal in cannabis concentrate users. In both concentrate and flower users, frequency of use is positively correlated with self-reported withdrawal and craving.[40] Our data show greater endorsement of withdrawal symptoms over the previous 12 months in concentrate users (*M*=3.2) vs. flower (*M*=2.4; *p* <.05), and that endorsement of withdrawal increases 15% after overnight abstinence affirming the link among THC reduction and withdrawal symptoms.

Effects of switching to cannabis with greater CBD content. In another study, we assigned 24 regular cannabis users to use either a high-THC cannabis strain similar to what they normally used (THC ~17%; CBD <= 1%) or a strain with lower THC and high CBD (THC ~8%; CBD ~16%).[41] After using their assigned strain on an *ad libitum* basis for 3 days, participants used the product again and were immediately transported to the laboratory for testing. Relative to participants who used the high-THC strain, those who used the lower THC/high CBD strain had a significantly lower desire to smoke more, felt less intoxicated, and demonstrated less impairment of verbal recall. These data indicate that adding CBD, even in the context of ongoing THC exposure, may mitigate some of the harmful effects of THC.

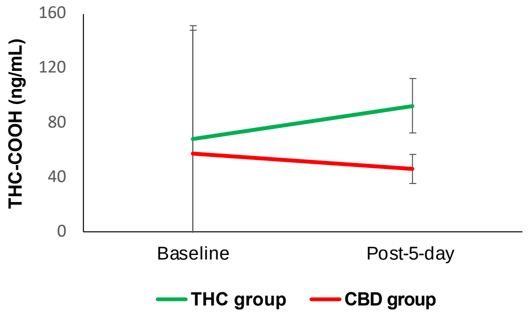

Figure 3. THC exposure, as assessed by THC-COOH levels, at baseline and following 5 days of a high-THC vs. high-CBD concentrate.

Effects of short-term CBD use on THC use and withdrawal in concentrate users. We conducted a preliminary naturalistic study on the effect of short-term CBD use in 54 concentrate

users.[6] Participants were randomly assigned to continue using a high-THC concentrate (85% THC, <1% CBD) similar to their typically used product (*n*=28) or to switch to a high-CBD concentrate (79% CBD, 4.5% THC) (*n*=26) for 5 days. Participants completed a baseline session, followed by a 5-day *ad libitum* use period and an experimental session evaluating the effects of naturalistic use of their assigned concentrate product. As compared to participants in the THC group, those in the CBD group demonstrated a significant reduction in THC exposure, as indexed by blood levels of the THC metabolite 11-nor-9-carboxy-THC (THC-COOH) (Figure 3). In addition, those assigned to the CBD-dominant concentrate reported a significant reduction in the number of withdrawal symptoms endorsed on the Marijuana Withdrawal Checklist (MWC) over the 5 day *ad libitum* period (MWC at Baseline: *M(SD)*=11.8(4.8) vs MWS after 5 days of CBD use (*M(SD)*=7.8(5.2)). Lastly, the THC-dominant concentrate acutely elicited higher intoxication, ratings of drug effect, and reward than the CBD-dominant concentrate. Immediately following use, the CBD-dominant group displayed decreased tension and anxiety, while the THC-dominant group demonstrated increased paranoia that persisted for an hour. Overall, these preliminary data suggest that CBD reduces THC exposure, withdrawal, anxiety, and negative affect in concentrate users.

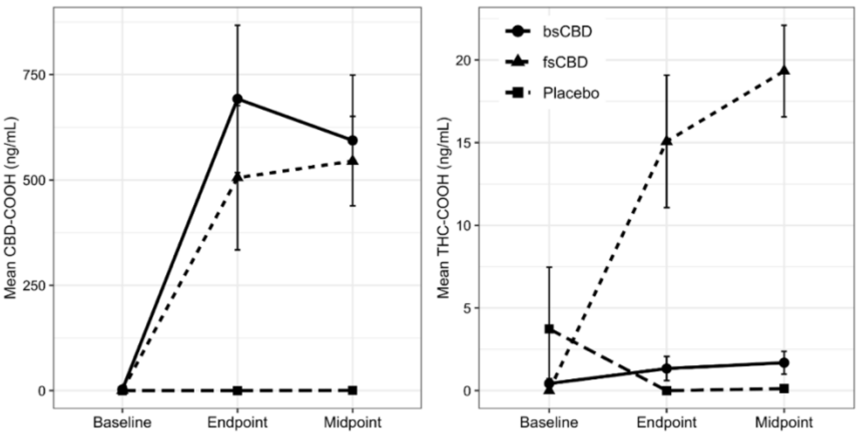

Figure 4. CBD-COOH and THC-COOH plasma levels across time in ongoing randomized controlled trails for hemp-derived CBD in substance use disorders by condition: fsCBD (n=21), bsCBD (n=19), and placebo (n=17). Study midpoints range from 4-6 weeks; study endpoints range from 8-12 weeks.

Ongoing clinical trials of hemp-derived CBD and substance use: Product compliance and cannabinoid biomarker levels. We currently have 4 FDA-approved trials underway examining the effects of hemp-derived CBD on health behaviors, including opiate and alcohol use disorders. Figure 4 summarizes cannabinoid biomarker data from these substance use-focused RCTs showing the changes in 7-Carboxy cannabidiol (CBD-COOH) and THC-COOH by treatment condition (bs CBD, fsCBD, and placebo) over the trial period. As expected, CBD metabolite levels increased significantly in the two treatment conditions compared to placebo over the treatment period. THC-COOH levels were marginally higher, but not significantly different in the fsCBD condition, as compared to the bsCBD condition. Note these data are in individuals with alcohol and opioid use disorders, not cannabis users. These data support the rigor of our hemp-derived RCT design, validate the constituent cannabinoids of our fsCBD and bsCBD products, and support the feasibility of extending these designs in the proposed research by comparing the effects of fsCBD, bsCBD, and placebo in cannabis concentrate users.

Medication Adherence. It is also important to note that medication adherence in these ongoing trials is 97% (i.e., 97% of doses taken) at the midpoint evaluation and 95% at end of treatment and adherence did not differ by condition, suggesting high levels of compliance.

fsCBD Reduces Cannabis Use. While cannabis use was neither an inclusion criteria nor an explicit treatment target of either of our ongoing trials, it is useful to note that the mean number of cannabis use days decreased within the fsCBD condition across the trial period (baseline mean cannabis use days (*M(SD)*=6.56(13.02); midpoint, *M(SD)*=5.56(11.3); endpoint,

M(SD)=3.75(10.67)), providing hypothesis consistent data supporting the potential of fsCBD to decrease cannabis use.

IV. RESEARCH STUDY DESIGN

Overview of study design.

To assess the effectiveness of an 8-week hemp-derived cannabidiol (CBD) intervention in reducing the craving and use of high potency THC products, participants will be randomly assigned to one of three groups: broad-spectrum CBD (bsCBD: containing 400mg CBD and 0% THC), full-spectrum CBD (fsCBD: containing 400mg CBD with ~0.3% THC), or placebo (0 mg CBD and 0% THC). Participants in the bsCBD and fsCBD groups will take 200mg (4 x 50mg softgels) in the morning and 200mg (4 x 50mg softgels) in the evening, for a total of 400 mg/day. Participants in the placebo group will take 4, 0mg CBD and 0% THC softgels in the morning and in the evening. Data addressing this study's primary and secondary aims will be collected from 8 study visits (6 in-person: Baseline, and Weeks 1, 2, 4, 6, and 8; and 2 telehealth: Weeks 12 and 16). **Baseline**: Informed consent will be obtained, inclusion/exclusion criteria, cannabis use, including quantity and frequency of THC and CBD use, CUD, and withdrawal will be assessed, and biological samples (blood and urine) will be collected. Participants will also participate in the first of five psychological intervention sessions after which participants will be randomly assigned to take bsCBD, fsCBD, or placebo daily for the next 8 weeks. **Weeks 1, 2, 4, and 6:** Softgel counts, Medication Event Monitoring Systems (MEMS) Caps data, and biological (blood at Weeks 1 and 4 and urine at all visits) samples will be collected to assess THC use and medication adherence from the previous visit; participants will self-report on withdrawal and THC use over the same period and complete the second, third, fourth, and fifth psychological intervention sessions, respectively. **Week 8:** This final in-person visit will include softgel counts, MEMS caps, biological, and self-report assessments from the previous visit. Use of study medication will cease after the completion of the week 8 visit. The final two visits at Weeks 12 and 16 will be telehealth follow-ups to assess cannabis use, withdrawal, and CUD at 4- and 8-weeks post-study medication cessation.

Measures.

Adverse events. Research assistants will query participants about any adverse events experienced.

Anthropometrics. Height and weight will be measured with a stadiometer and scale. Waist circumference and hip circumference will be measured with a measuring tape.

Anxiety, Depression, and Suicidality. The Depression Anxiety Stress Scale (DASS) [42] consists of 21 items designed to measure the emotional states of depression, anxiety, and stress in three subscales. The Columbia-Suicide Severity Rating Scale (C-SSRS) [43] includes six items that categorizes an individual as low, moderate, or high risk for suicidality.

Breathalyzer. The breathalyzer will be used to measure breath alcohol level.

Cannabis use disorder, cannabis withdrawal symptoms, and cannabis craving. The Marijuana Dependence Scale (MDS)[44] is based on DSM V criteria that were converted to a self-report measure. Individuals report on each dependence item and items are then summed to form the scale (α=.85). This scale is validated and has been previously used in the cannabis literature. The Cannabis Use Disorders Identification Test – Revised (CUDIT-R)[45] consists of 8 items designed to identify potentially problematic or harmful recent cannabis use. The Marijuana Withdrawal Checklist (MWC) [46], will assess 15 cannabis withdrawal symptoms. The Cannabis Craving Scale will assess cannabis craving[47].

Clinical Blood Labs. Clinical labs will include a comprehensive metabolic panel (CMP) to assess liver function and a complete blood count (CBC) to assess general health.

Demographics including age, sex assigned at birth, gender identity, sexual orientation, marital status, race and ethnicity, socioeconomic status, occupation/retirement status, income, education, and neighborhood (zip code and county) will be assessed.

Effect expectancies. To assess and control for differences in cannabis effect expectancies between participants, participants will complete the Marijuana Effect Expectancy Questionnaire - Brief [48] and the Cannabis Effect Expectancy– Medical (CEEQ-M) [49].

General Health. NIH's Patient Reported Outcomes Measurement Information System (PROMIS) Global Health survey includes 10 questions to assess an individual's physical, mental, and social health.

Medical and psychiatric history. Research assistants will ask participants about their medical and psychiatric history. In addition, research assistants will conduct the Mini-International Neuropsychiatric Interview (MINI) to assess CUD diagnostic criteria and psychiatric comorbidity. PI Bidwell, a licensed clinical psychologist, will supervise the training of research assistants in the conduct of the MINI to ensure competency.

Medication adherence. MEMS caps will be used to collect data about frequency, time, and date of bottle openings. Manual softgel counts will also be conducted.

Physical exam and vital signs. Heart rate, blood pressure, and oximetry on room air will be measured. A physical exam will be conducted by a physician at the CTRC to assess general health.

Subjective measures of substance use. A Substance Use History Questionnaire (SUHQ) will assess frequency of lifetime and recent illicit drug use. Current **medications** will also be tracked. The Daily Sessions, Frequency, Age of Onset, and Quantity of Cannabis Use Inventory (DFAQ-CU)[50] is used to collect information on the frequency and quantity of cannabis use, age of first use, peer use, perceived risk from cannabis, and perceived availability of cannabis. The Alcohol Use Disorder Identification Test (AUDIT)[51] will be used to examine the extent of alcohol use and problems related to alcohol use. A Timeline Follow-Back (TLFB)[52] will be used to assess daily substance use. The TLFB is a calendar assisted tool that provides the subject with temporal cues to increase the accuracy of recall. This instrument has demonstrated test-retest reliability and validity[53]. The TLFB also records alcohol use, tobacco use, use of illegal drugs, and recreational use of prescription drugs such as anti-depressants, Adderall, Ritalin, and Vicodin. We have modified our TLFB procedure to estimate in detail the frequency, type, amount, and potency of cannabis use each day[54]. Participants will also be asked a question about frequency of Driving After Cannabis Use.

Objective measures of substance use. A breathalyzer will ensure a breath alcohol concentration of zero at the beginning of each session. We will conduct urine testing for recent use of drugs of abuse other than cannabis using an on-site Syva Rapid Test (Dade-Behring, San Jose, CA).

Sleep. NIH's Patient Reported Outcomes Measurement Information System (PROMIS) Sleep Disturbance and Sleep-Related Impairment measure will assess sleep functioning.

THC exposure. Each blood sample will be assayed using HPLC-MS-MS for cannabinoids (inter-assay precision is within 85-115% and total imprecision, except at lower limit of quantification, is better than 15%). The cannabinoid and endocannabinoid assays utilized in the iC42 laboratory have been completely validated following FDA guidelines for bioanalytical method development, and the methods are published.[55] Urine samples will also be collected and assayed to quantify THC-COOH.

Urine pregnancy. Urine pregnancy tests will be conducted to ensure female participants are not pregnant.

Name of procedure/instrument/tool	Purpose (i.e., what data is being collected?)

Self Report/Interview Measures	
Alcohol Use Disorder Identification Test (AUDIT)[56]	Examines the extent of alcohol use and problems related to alcohol use
Cannabis Craving Scale (CCS)[47]	Assesses cannabis craving
Cannabis Effect Expectancy– Medical (CEEQ-M) [49]	Evaluates expectancies for cannabis used for medical symptoms
Columbia-Suicide Severity Rating Scale (C-SSRS)[43]	Assesses suicidality
Cannabis Use Disorder Identification Test – Revised (CUDIT-R)[45]	Assesses problematic cannabis use
Daily Diary	Assesses cannabis use, craving[47], and withdrawal symptoms via emailed REDCap survey
Demographics	Includes age, sex assigned at birth, gender identity, sexual orientation, marital status, race and ethnicity, socioeconomic status, occupation/retirement status, income, education, and neighborhood (zip code and county)
Depression Anxiety Stress Scale (DASS)[42]	Measures the three related negative emotional states of depression, anxiety, and tension/stress with 12 items
Daily Sessions, Frequency, Age of Onset, and Quantity of Cannabis Use Inventory (DFAQ-CU)[50]	Measures frequency and quantity of cannabis use, as well as age of first use
Driving After Cannabis Use	A single question assessing the frequency of driving within two hours of cannabis consumption
Marijuana Dependence Scale (MDS)[60]	Assesses CUD based on DSM-V criteria
Marijuana Effect Expectancy Questionnaire – Brief (MEEQ-B)[48]	Measures cannabis effect expectancies
Marijuana Withdrawal Checklist (MWC)[46]	Assesses 15 cannabis withdrawal symptoms
Medical and Psychiatric History	Brief interview for significant past illnesses, surgeries, and psychiatric diagnoses
Mini-International Neuropsychiatric Interview (MINI)	Structured interview to assess psychiatric and CUD diagnostic criteria
Patient Reported Outcomes Measurement Information System (PROMIS) Global Health	Assesses physical, mental, and social health.
Patient Reported Outcomes Measurement Information System (PROMIS) Sleep Disturbance and Impairment	Weekly sleep functioning
Prescription Medication Review	Research assistant will record dose, frequency, and reason for use for all medications
Substance Use History Questionnaire (SUHQ)	Frequency of lifetime and recent use for cocaine, amphetamine, opiates, sedatives, and hallucinogens
Timeline Follow-Back (TLFB)[61]	Assesses daily substance use for the 30 days prior to interview, specialized for cannabis use and consistent with the validated Cannabis Exposure Inventory[62] and NIDA guidelines to reduce concentrate use by standard units (5mg THC).

Objective Health and Biomarkers	
Anthropometrics	Height, weight, waist circumference, hip circumference
Breathalyzer	Assesses breath alcohol levels
Blood Levels of Cannabinoids	Blood levels of THC, 11-OH-THC, THC-COOH, THC-COO-glucuronide, THC-glucuronide, CBD, 6α-OH-CBD, 6β-OH-CBD, 7-OH-CBD, CBD-COOH, CBD-glucuronide, CBN, CBC, CBDV, and THCV
Complete Blood Count (CBC)	Measure of general health (e.g., red blood cells, white blood cells, platelets)
Comprehensive Metabolic Panel (CMP)	Measure of liver function (e.g., alanine aminotransferase (ALT), aspartate aminotransferase (AST))
Softgel count and Medication Adherence Packaging (MEMS) cap data [63,64]	Manual study drug softgel count and softgel bottle use capture metrics (i.e., time/date/frequency of bottle opening)
Physical exam	Assesses general health
Plasma CBD-COOH	Quantitation of blood CBD metabolite levels to evaluate medication adherence
Urine Pregnancy Test	Used to confirm participants assigned female at birth are not pregnant during study participation
Urine THC-COOH	Quantitation of urine THC metabolite levels to evaluate recent cannabis use
Urine Toxicology	Used to determine recent use of cocaine, opiates, methamphetamine, MDMA, benzodiazepines, or barbiturates
Vital signs	Blood pressure, heart rate, oximetry

Randomization.

Participants will be randomized to either 400 mg fsCBD, 400 mg bsCBD, or placebo in a 1:1:1 fashion once eligibility is confirmed by the study physician. A pre-determined randomization table developed by the study statistician (Co-I Dr. Bryan) will be used and both study staff and participants will remain blind to group assignment throughout the study. A study coordinator (Gregory Giordano) not involved in data collection will maintain the blind.

Power and Data Analysis

*Sample size determination and power analysis***.** Since power is determined by the analysis that requires the largest number of participants (Exploratory Aim 3), we based our power analysis on the mediational model proposed in Exploratory Aim 3 (see Figure 6 below). Based on our prior work and review of the literature, we expected small to moderate coefficients for most paths in the mediational models, with parameter estimates in the range of .30 to .35 for paths from the active medication versus placebo contrast to the mediators and from the withdrawal mediators to THC use. We estimated somewhat smaller parameter estimates of .25 from the fsCBD vs bsCBD contrast to the mediators. Power analyses were conducted in Mplus and then in SAS following procedures for estimating the power of the likelihood ratio test of the significance of parameters in structural equation models.[65] We utilized Monte Carlo simulation to generate a population covariance matrix based on the hypothesized parameters in the model. We evaluated power at a range of sample sizes. For the smallest path coefficient in the model, i.e., that between the fsCBD vs bsCBD contrast to the mediators, assuming two-tailed alpha of .05, a sample size of 100 gave us only .63 power, a sample size of 125 gave us .73 power and at sample size of 150 we have .80 power. For each of the larger paths in the model, we have over .91 power with 150 participants.

Anticipated attrition. Based on our previous studies with this population, we anticipate a 10% attrition rate between the baseline session and the week 8 session. We have not previously experienced and do not anticipate differential attrition by medication group. (Proportion of completers/enrolled in our current 8 week Alcohol Use Disorder CBD trial: 11/12 bsCBD group; 10/12 completed fsCBD group, 11/12 competed placebo group). Thus, we will recruit a total of 55 per medication group (n=165) with a target of n=150 complete participants. Our approach to power analysis and accounting for attrition is extremely conservative, in that the techniques we will utilize in data analysis use state of the art recommendations in iterating the estimation of missing data.[66] All analyses are intent-to-treat and all available data are utilized.

Statistical analysis plan. Analyses will be conducted with SAS v.9.4 (SAS Institute, Cary, NC) or R (R Core Team, 2020), which both include capabilities to test multilevel models that include nonlinear effects and missing data. All aims will be tested with a multilevel modeling framework to account for the repeated observations nested within participants over time. Preliminary analyses will evaluate all data for normality and reliability. Three-group ANOVAs and χ^2 tests will be used to compare baseline variables (e.g., age, sex, race, AUDIT, depression and anxiety symptoms, cannabis expectancies) between groups. Characteristics that differ between groups will be evaluated as potential covariates in subsequent analyses. Given the intentionally broad age range, age will be covaried in all analyses, and age by group interactions will be tested in sensitivity analyses to determine whether the effects of hemp-derived CBD differ in older vs. younger individuals. Pairwise post-hoc simple effects tests will be conducted to confirm patterns of differences. These analyses for demographic differences will be controlled for Type I error inflation using false discovery rate correction.[67]

Aim 1: Test the effect of fsCBD and bsCBD, relative to placebo, on THC use and CUD over 8 weeks. The primary outcomes for Aim 1 are: 1) THC use during the study period as assessed by self-reported total THC mg based on our modified TLFB that allows detailed quantitation of quantity of cannabis use, including the average number of standard 5 mg THC units used per day, across the 8 week study, 2) THC use during the study period, as assessed by participants' urine THC-COOH levels (standardized for creatinine) from the baseline, week 1, 2, 4, 6, and 8 visits; and 3) CUD symptoms at baseline and 8 weeks. One linear mixed model that includes group (two orthogonal contrast codes: 1) fsCBD and bsCBD vs. placebo and 2) fsCBD vs bsCBD), time, and their interactions will be tested for each outcome, with the critical term being the interaction between each group contrast code and time. For THC use, we predict an interaction between group and time, such that the fsCBD and bsCBD, relative to placebo, will display lower levels of THC use (as measured by average number of standard 5 mg THC units / day) at weeks 1, 2, 4, 6, and 8 and fsCBD versus bsCBD will display lower THC use over time. We predict the same effects for urine THC-COOH levels across the 8 week study and for CUD at 8 weeks.

Aim 2: Test the effect of fsCBD and bsCBD, relative to placebo, on affective, physiological, and physical withdrawal symptoms. The primary outcomes for Aim 2 are: 1) total withdrawal symptoms measured by the MWC at 1, 2, and 4 weeks and 2) the three subfacets of withdrawal including affective, physiological, and physical withdrawal symptoms measured at 1, 2, and 4 weeks. Similar to Aim 1, one linear mixed model that includes the 2 orthogonal contrasts for group, time, and their interaction will be tested for each outcome, with the critical term being the interactions between each group contrast code and time. The same effects are predicted as above, such that fsCBD and bsCBD, relative to placebo will display lower levels of withdrawal at weeks 1, 2, and 4, and the fsCBD relative to bsCBD will display lower levels of withdrawal.

Aim 3: Explore whether the effect of fsCBD and bsCBD, relative to placebo, on reducing THC use is mediated by reduction in withdrawal. To test mediation, the same two orthogonal contrast codes for group will serve as exogenous variables in a path analytic mediational model consistent with procedures utilized by Co-Bryan in previous studies.[68] As visualized in Figure 6, the three mediating variables are changes in affective, physiological, and physical withdrawal symptoms represented by change scores from week 1 to week 4. The outcome variable is total mg of THC used over the 8 week study. (Week 8 THC-COOH urine levels and week 8 CUD symptoms will be tested as secondary outcomes in this exploratory mediation model using the same framework.) This model will be estimated and both the fit of the model and the significance of the path coefficients will be examined. If the paths from the medication group to changes in withdrawal symptoms and from changes in withdrawal symptoms to amount of THC use are significant, then mediation is suggested. A test for completeness of mediation is employed through a series of 1 degree of freedom $\chi2$ tests where a path directly from group contrast to week 8 total THC use is added to the model. A nonsignificant direct path and a nonsignificant change in $\chi2$ suggest that medication effects on the outcome were mediated through changes in withdrawal.[69] A secondary test of mediation will utilize bootstrap methods to test the significance of, and confidence limits around, the mediated effect.[65,70] We predict that changes in withdrawal will at least partially mediate the effect of hemp-derived CBD on reduced THC use.

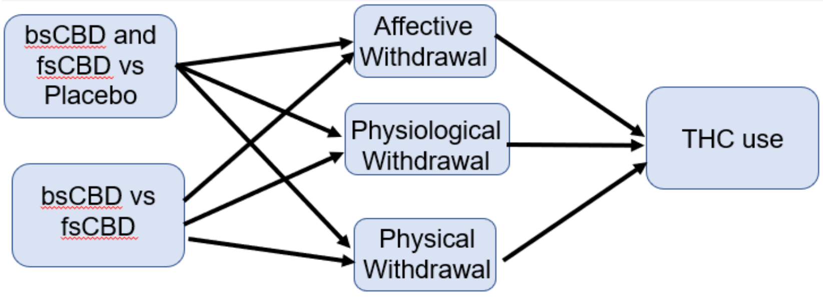

Figure 6. Hypothesized relationships between hemp-derived CBD and reductions in THC use via changes in withdrawal symptoms. The mediator (withdrawal) is measured part way through treatment (weeks 1 to 4), while outcome (THC use) is measured at the end of 8 weeks of

Exploratory analysis of sex differences in THC effects and cannabinoid metabolism. Recent preclinical and clinical data suggest that THC effects may differ by sex, and that this disparity may be related to differences in cannabinoid metabolism. Specifically, following high-dose (5 mg/kg) THC administration, female rodents displayed markedly different THC metabolism than males, characterized by elevated levels of the primary active cannabinoid metabolite 11-hydroxy-THC (11-OH-THC), which itself is neuroactive and binds to cannabinoid receptors.[71] Further, although the prevalence of use is lower among females, female users develop CUD at a faster rate as compared to men (a finding known as the “telescoping effect”).[72] Females also have an increased rate of cannabis withdrawal and report greater severity of certain withdrawal symptoms compared to men.[73] These differences may potentiate and/or prolong the effects of high dose THC, CUD, and withdrawal in females. Consistent with these findings, a recent clinical study of low-to-medium (5-25 mg) THC doses among infrequent cannabis users found that female participants, relative to males, displayed greater 11-OH-THC concentrations (replicating the preclinical effect) and reported greater drug effects after THC administration.[74] Thus, women may be particularly adversely affected by high-dose THC use. To assess the effect of sex as a biological variable, sex will be tested as an exploratory moderator of all group effects analyzed in Aims 1-3; since equal numbers of male and female participants will be recruited, and the *N* is sufficient to detect small effect sizes, these analyses will also be adequately powered. If women are especially impacted by products that facilitate heavy THC use, this would be critical public health information for policy makers and the public.

V. FUNDING

This study is funded by a grant to the University of Colorado Boulder (1 R01 DA059234) from the National Institute on Drug Abuse (NIDA).

VI. ABOUT THE RESEARCH PARTICIPANTS

We will recruit a sample of 165 participants who are heavy, stable cannabis concentrate users that meet criteria for at least mild CUD and are seeking to cut down or stop their use. Participants will be 21 years or older (further eligibility details are included in the table below). Participants will be pre-screened via an online eligibility form or through the phone by a trained research member for age, cannabis use history, CUD, desire to cut down or stop cannabis use, medication use, use of CBD-dominant products, alcohol use, nicotine use, psychiatric history, and medical history. Blood tests for liver functioning will be conducted and all other eligibility criteria will be reviewed at the Baseline. The study physician will review all results from the Baseline to determine continuing eligibility prior to participants being randomized and receiving study medication.

**Inclusion criteria**
Ages 21 and over.
Regular use (at least 4 times per week) of cannabis concentrates for at least the last year.
Meets DSM5 criteria for at least moderate CUD (4 or more symptoms).
Currently seeking to cut down or stop cannabis use.
**Exclusion criteria**
Use of any substance of abuse besides alcohol, nicotine, or cannabis (e.g., cocaine, non-prescription use of opiates, methamphetamine, MDMA, benzodiazepines, or barbiturates) in the past 90 days, as indicated by self-report and urine toxicology screening (Syva Rapid Test) at baseline.
Use of CBD-dominant products in the past 90 days, as evidenced by self-report of use of a CBD>THC product or CBD blood levels at baseline of >= 5 ng/mL which is based on the low CBD plasma levels typical of high potency concentrate users[75].*
Alcohol use on 3 or more days per week, and/or > 3 drinks per drinking day in the past 90 days. Participants must also have a breath alcohol level of 0 at the beginning of each study visit.
Daily nicotine use.
Meets DSM-5 diagnostic criteria for a psychotic disorder (e.g., schizophrenia, schizophreniform disorder, schizoaffective disorder), bipolar disorder, or major depression with suicidal ideation, or has a history of treatment for these disorders. Psychiatric disorders will be assessed with the Mini-International Neuropsychiatric Interview (MINI).
Current cardiovascular or respiratory disease (e.g., coronary artery disease, severe asthma, chronic obstructive pulmonary disease, etc.)
Current use of psychotropics (e.g., antidepressants, anxiogenics), which may dampen effects of CBD.
Currently use of anti-epileptic medications (e.g., clobazam, sodium valproate) or medications known to have major interactions with Epidiolex (buprenorphine, leflunomide, levomethadyl acetate, lomitapide, mipomersen, pexidartinib, propoxyphene, sodium oxybate, and/or teriflunomide).
Current or past hepatocellular disease, as indicated by alanine aminotransferase (ALT) or aspartate aminotransferase (AST) > 2 times the upper limit of the normal range at screening or a history of liver disease irrespective of AST and ALT at the time of screening.
For participants assigned female at birth, breastfeeding, pregnancy, or trying to become pregnant. A positive urine pregnancy test at the beginning of any study visit will result in exclusion from ongoing study participation.
History of seizures
Current use of potent CYP2C19 or CYP3A4 inducers (e.g., Rifampin, apalutamide, carbamazepine, enzalutamide, ivosidenib9, lumacaftor, ivacaftor, phenytoin, St. John's wort, Fosphenytoin, Mitotane, Phenobarbital, Primidone), or strong CYP3A inhibitors (e.g., clarithromycin, HIV protease inhibitors, and most

antifungals), 2C19 inhibitors (e.g., fluoxetine, Lansoprazole, Tricyclic antidepressants (TCAs))
Allergy to study medication ingredients (hemp seed oil, hemp extract, gelatin, glycerin)

Participant Population(s)	Number to be enrolled in each group
400 mg fsCBD	55
400 mg bsCBD	55
Placebo	55

VII. VULNERABLE POPULATIONS

This study does not include any vulnerable populations.

VIII. RECRUITMENT METHODS

Participants will be recruited from the greater Boulder/Denver metropolitan area. These individuals will be recruited via a number of sources that have been used successfully by our research team to recruit cannabis concentrate users. First, as in all our other cannabis projects to date, we will recruit using flyers posted in dispensaries and ads on the webpages and social media pages of dispensaries and other cannabis-related businesses and organizations in the Denver area. Second, we will utilize targeted mailings advertising the opportunity to participate in our study. For this recruitment method, we will obtain a list of names and addresses of individuals who fit our target demographics (age, gender, and other criteria) and geographical area using information obtained from publicly available records purchased from a marketing firm). We will also use social media advertising that allows for targeting ads based on age, geographic location, and interests (in this case, following or liking posts related to cannabis). Finally, to achieve our goals for recruiting diverse participants, focused efforts will be undertaken which leverage a community engaged research approach to recruitment. Community engaged research recruitment strategies are generally more interpersonal in nature than traditional strategies, including development of content-specific presentation materials to be shared with potential partner groups and key stakeholders during community events, town halls, recurring meetings, or leadership sessions. Community-engaged recruitment also involves the planning and coordination of targeted recruitment events and follow-up with both potential participants and key stakeholders from partnering organizations. All recruited participants will be engaged in an informed consent process with study personnel.

Individuals interested in participating will be given the option of completing a pre-screening questionnaire over the phone or via an online screening form. The online screening form will also be accessible via email or a QR code placed on recruitment materials. Trained research personnel will review the responses of prospective participants according to the inclusion/exclusion criteria in Table 1 related to age, cannabis use history, CUD, desire to cut down or stop cannabis use, medication use, use of CBD-dominant products, alcohol use, nicotine use, psychiatric history, medical history, and allergies to study medication ingredients. If anyone is questionable for inclusion at this point, the study physician will make the final determination. Individuals completing the pre-screen over the phone will be informed of preliminary eligibility status over the phone at the time of the pre-screen. Individuals completing the online screening form will be informed of preliminary eligibility status over the phone if eligible, or via email if found ineligible within 7 days of completing the pre-screen. Participants meeting the inclusion/exclusion criteria assessable by the pre-screen will be scheduled for their

Baseline visit and will be instructed not to drink alcohol for 24 hours and to not use caffeine or nicotine 1 hour prior to the session.

Recruitment Methods
**1.** Flyers and business cards to be distributed in dispensaries
2. Community-engaged coordination with key stakeholders (e.g., presentations at community events, town halls, leadership sessions)
**3.** Mailing lists using marketing firm services and age specific institutions (e.g., senior community centers)
**4.** Social media advertising across social media platforms (e.g, Facebook)
5. Advertisements placed on cannabis-related business websites

IX. COMPENSATION

Participants will receive up to $390 for completing all aspects of the study; $80 for completing the Baseline visit, $40 for completing Week 1, $40 for completing Week 2, $60 for completing Week 4, $40 for completing Week 6, and $60 for completion of the final study visit at Week 8. Participants will also receive $20 for each of the follow-up telehealth appointments (Week 12 and 16). Participants will receive $15 for completing at least 80% (23 of 28) of scheduled daily diaries between Week 1 and Week 4 visits, and $15 for completing at least 80% (23 of 28) of scheduled daily diaries between Week 4 and Week 8 visits. All payments for study appointments will be in cash at the conclusion of each visit, payments for daily diaries will be made in cash at the conclusion of the Week 4 and Week 8 visits, and payments for telehealth appointments will be made with gift cards from a selection of retailers emailed to participants.

X. INFORMED CONSENT

When a participant arrives for their first visit at the Clinical Translational Research Center (CTRC), a member of the research team will greet them in the lobby (note that ample free parking is available for research participants at the CTRC). The trained research assistant will take the participant to a private room and show the participant a copy of the informed consent document. Prior to asking the participant to sign the consent form, the research assistant and the participant will have a discussion regarding the research study during which the research assistant will answer any questions participants may have about the study. Participation will be clearly stated as voluntary, with the option to withdraw from the study at any time. Should participants choose to participate, they will sign the informed consent document. The research assistant obtaining informed consent will also sign the informed consent document to document the informed consent process. Participants will be given a copy of the signed informed consent document.

XI. PROCEDURES

Baseline. The Baseline will be conducted by a research assistant in a private room at the Clinical Translational Research Center (CTRC) on the University of Colorado Boulder campus. Before the Baseline, participants will be instructed not to consume caffeine or nicotine for one hour prior or drink alcohol for 24 hours prior to their visit. This visit will be held in the morning. After providing informed consent, participants will blow into a breathalyzer device to ensure that

they have not recently used alcohol. Participants with a breath alcohol level above 0 will be dropped from the study. A urine sample will be collected for a toxicology screen, to quantify cannabinoids, and to conduct a pregnancy test for females. Participants testing positive for substances other than cannabis, nicotine, or alcohol will be dropped from the study. Pregnant individuals will be dropped from the study. Participants will be instructed to use an effective form of birth control until use of the study medication is complete. A blood draw will be obtained for clinical chemistry to ensure that participants do not have laboratory abnormalities that are contraindications for CBD use (e.g., impaired liver function), as well as for cannabinoid quantitation. Height, weight, waist circumference, hip circumference, blood pressure, heart rate, and oximetry on room air will be measured. A physical exam will be conducted by a physician at the CTRC. Medical and psychiatric history, as well as current medications will be collected by the research assistant. The research assistant will also conduct the MINI, a structured interview to assess psychiatric and substance use disorders. The MINI will be used to assess CUD symptoms to determine participant eligibility. The participant will then be provided with an iPad to complete REDCap surveys on demographics, general health, substance use, cannabis withdrawal symptoms, anxiety, depression, suicidality, sleep, and expectancies about cannabis use.

The study physician will review all results from the Baseline visit (e.g., medical history, medications, CBC, CMP) to determine continuing eligibility for each participant. Results from the MINI will also be presented to the physician to determine eligibility. The research assistant who conducted the interview and PI Bidwell (a licensed clinical psychologist) will be available to consult on the MINI results, if needed. PI Bidwell will also review and sign off on the results of the MINI. Participants found to still be eligible will be randomized to either CBD+THC, CBD, or placebo conditions based on a pre-generated random selection list and scheduled for the first of five remote therapy sessions.

Daily Diaries. Participants will be emailed a brief REDCap survey daily to record any cannabis use, craving[47], and withdrawal symptoms between the Baseline and Week 8 visits.

Psychological Intervention. Participants in both experimental groups and the control group will all participate in five telehealth-based psychotherapy sessions that cover the initial components (cognitive behavioral therapy [CBT] Skill Topics 1-6) of the Substance Abuse and Mental Health Services (SAMSHA) treatment protocol to support motivation for cannabis use reduction and provide standard of care to all participants. Each psychotherapy session will take place within five days of a participant's corresponding study visit and will be scheduled directly with the study therapist (e.g., Session 1 with Initial Medication Dispense, Session 2 with Week 1 Visit, Session 3 with Week 2 Visit, Session 4 with Week 4 Visit, Session 5 with Week 6 Visit). Study therapists will meet one-on-one with participants via Zoom during these sessions.

Initial Medication Dispense. Eligible participants will be scheduled for an Initial Medication Dispense Visit at CUChange to receive 2 weeks of medication. A trained research assistant will provide the participant with the dosage regimen and administration instructions. Specifically, participants in all conditions will be asked to take 200mg of their assigned product twice a day via four softgels (i.e., four softgels once in the morning and four softgels once in the evening), for a total daily dose of 400mg of CBD in both the CBD+THC and CBD groups, or 400mg of Hemp Seed Oil in the placebo group (see **Hemp-derived CBD product and dosing in Drug Administration).** Participants will be instructed to take the study medication with food. Notably, this is a double-blind trial, such that both the researcher conducting the study assignment and the participant are blind to condition. Each condition will simply be labeled with a letter (X, Y, or Z) and a member of the team not involved in data collection will maintain the blind.

Weeks 1, 2, 4, & 6 . Participants will meet with a research assistant at CUChange one, two, four, and six weeks after receiving their study medication. Participants will be instructed not to consume caffeine or nicotine for one hour prior or drink alcohol for 24 hours prior to each visit. Each visit will be held in the morning. Participants will also be instructed to bring their medication bottle to each visit so that the MEMS cap data can be collected and the number of softgels

remaining can be counted, and to wait to take their first dose for the day until after the appointment is complete. Breath alcohol level will be tested and participants with a breath alcohol level above 0 will be dropped from the study. A urine sample will be collected for to quantify cannabinoids and to conduct a pregnancy test for females. Pregnant individuals will be dropped from the study. A blood draw will be obtained to measure cannabinoids at Weeks 1 and 4. Current medications and any adverse events experienced by the participant will be recorded by the research assistant. The participant will then be provided with an iPad to complete REDCap surveys on substance use, sleep, cannabis craving, and withdrawal. Participants will receive additional medication at Week 1, Week 2, Week 4, and Week 6. The Week 4 Visit will also include a liver function test (taken from the blood draw), a suicidality survey, and measurement of weight, waist circumference, hip circumference, blood pressure, heart rate, and oximetry on room air.

Week 8. Participants will meet with a research assistant at CUChange eight weeks after receiving their study medication. Participants will be instructed not to consume caffeine or nicotine for one hour prior or drink alcohol for 24 hours prior to the visit. The visit will be held in the morning. Participants will also be instructed to bring their medication bottle to the visit so that the MEMS cap data can be collected and the number of softgels remaining can be counted, and to not take the study medication on the day of the visit. Any remaining study medication will be returned. Breath alcohol level will be tested and participants with a breath alcohol level above 0 will be dropped from the study. A urine sample will be collected for a to quantify cannabinoids and to conduct a pregnancy test for females. A blood draw will be obtained to measure cannabinoids and to check liver function. Weight, waist circumference, hip circumference, blood pressure, heart rate, and oximetry on room air will be measured. Current medications and any adverse events experienced by the participant will be recorded by the research assistant. The participant will then be provided with an iPad to complete REDCap surveys on general health, substance use, cannabis craving, cannabis withdrawal symptoms, anxiety, depression, suicidality, and sleep.. At the end of this visit, participants will receive additional debriefing regarding their study participation. Self-help resources will be offered and the range of continued treatment services available will be explained. If a participant wishes to pursue additional treatment, an appropriate referral will be made that day or at any future time that the participant desires.

Weeks 12 & 16. Although use of the study medication ends after the Week 8 Visit, participants will meet with a research assistant remotely (via Zoom) twelve and sixteen weeks after starting their study medication. Current medications and any adverse events experienced by the participant will be recorded by the research assistant. The participant will also complete REDCap surveys on substance use and mood using the device of their choice

Visit #	Procedures/Tools	Location	How much time the visit will take
**Visit 1: Baseline**	• Informed consent;<br>• Breathalyzer<br>• Urine collection (pregnancy test, toxicology, cannabinoids)<br>• Blood collection (liver function, cannabinoids)<br>• Anthropometrics<br>• Vital signs<br>• Physical exam<br>• Interviews (medical and psychiatric history, MINI, medications)	Clinical Translational Research Center (CTRC)	~2 hours

	• Surveys of demographics, substance use (SUHQ, AUDIT, OTLFB, DFAQ-CU, Driving After Cannabis Use), anxiety and depression (DASS), cannabis use disorder (MDS, CUDIT-R, MWC), suicidality (C-SSRS), general health (PROMIS Global Health), sleep (PROMIS sleep), and effect expectancies (MEEQ-B, MEEQ-M)		
**Visit 2: Initial Medication Dispense**	• Random assignment to condition after study physician approval<br>• Medication instructions and dispense<br>• Remote psychotherapy session #1 (within 5 days of Initial Medication Dispense)	CUChange space at CINC<br>+<br>Zoom (psychotherapy session)	~0.5 hour<br>+<br>50 minute psychotherapy session
**Visits 3-6: Weeks 1, 2, 4, & 6**	• MEMs cap/study medication count<br>• Breathalyzer<br>• Urine collection (pregnancy test, cannabinoids)<br>• Blood collection (cannabinoids (only Weeks 1 and 4), liver function (only Week 4))<br>• Anthropometrics (only Week 4)<br>• Vital signs (only Week 4)<br>• Interviews (adverse events, medications)<br>• Surveys of substance use (OTLFB, Driving After Cannabis Use (only Week 4)), cannabis craving (CCS), cannabis use disorder (MWC), sleep (PROMIS sleep), and suicidality (C-SSRS; only week 4)<br>• Remote psychotherapy sessions #2-5 (within 5	CUChange space at CINC<br>+<br>Zoom (psychotherapy sessions)	4 X ~1 hour (in-person visits)<br>+<br>4 X 50 minutes (psychotherapy sessions)

	days of corresponding in-person visit)		
**Visit 7: Week 8**	• MEMs cap/study medication count<br>• Breathalyzer<br>• Urine collection (pregnancy test, cannabinoids)<br>• Blood collection (cannabinoids, liver function)<br>• Anthropometrics<br>• Vital signs<br>• Interviews (adverse events, medications)<br>• Surveys of anxiety and depression (DASS), substance use (AUDIT, OTLFB, Driving After Cannabis Use), cannabis craving (CCS), cannabis use disorder (MWC, CUDIT-R, MDS), sleep (PROMIS sleep), general health (PROMIS Global Health), and suicidality (C-SSRS)<br>• Debriefing, referrals, and resources	CUChange space at CINC	~1 hour
**Visits 8 & 9: Weeks 12 & 16**	• Interviews (adverse events, medications)<br>• Surveys of anxiety and depression (DASS), substance use (AUDIT, OTLFB), cannabis use disorder (MWC, CUDIT-R, MDS), sleep (PROMIS sleep), general health (PROMIS Global), and suicidality (C-SSRS)	Zoom	~0.5 hour each
**Total time: 4 months**	**Participants earn up to $360**		**~13.5 hours**

XII. SPECIMEN MANAGEMENT

Blood and urine samples collected will be kept on ice or in a laboratory refrigerator until transfer to locked freezers at the CTRC or in the PI's laboratory. Urine samples and blood samples for cannabinoid analysis will be coded with a participant ID number. Blood samples for clinical measures (CBC and CMP) will be coded with the participant's name and date of birth. All

samples will remain in locked freezers until analysis by CUChange trained personnel or transportation to the University of Colorado Denver Anschutz Medical Campus or to Boulder Community Hospital. The coded samples designated for analysis of cannabinoid content will be shipped to the iC42 Clinical Research and Development Lab on dry ice. Cannabinoid results are received as a csv shared through a REDCap project. The blood samples for clinical measures (CBC and CMP) will be transported to the Boulder Community Hospital Laboratory by research personnel or by a medical courier. Individual results from the Boulder Community Hospital Laboratory are sent via fax or mail. After all analyses are complete, all specimens will be destroyed.

XIII. DATA MANAGEMENT

The Data Classification is Confidential with a Moderate Adverse Impact Level. Participant confidentiality is strictly held in trust by the PI and all other key study personnel, thus a standard operating procedure for the management of participant data is in place across the PIs current and previous studies. Signed informed consent forms and any physical data will be stored in a locked filing cabinet in the PI's lab at the CINC. Physical data received from Boulder Community Hospital (CBC and CMP) with participant identifiers present will be stored in a locked filing cabinet separate from their participant IDs. Only the PI and key study personnel will have access to the cabinet. All coded data will be entered into REDCap, a secure, HIPAA-compliant, 21 CFR Part 11-ready data capture system provided by the University of Colorado Denver. REDCap is a centralized data management strategy that includes password protection and internal quality checks including dedicated accounts for each use, automatic log-off, required annual password updates for all users, granular-level user access settings, REDCap administrator project review to ensure proper handling of identifying data, and encrypted and secure storage provided and maintained by the Colorado Clinical and Translational Sciences Institute (https://redcap.ucdenver.edu/). REDCap is organized into discreet, unlinked units called "projects" that will be utilized variously for the management and storage of participant data and identifying information. Self-report data will be entered manually directly by participants responding to self-report measures in REDCap. There is no intermediary "intake form" or other collection tool, thus minimizing paper forms and helping to secure participant information. The master list linking participant IDs to identifying information will be stored in a discreet REDCap project. This project will only be accessible by research team members via their personal REDCap credentials, and only the PI will have "user rights" to assign team members to study related projects in REDCap. Thus, REDCap projects containing identifying information are never linked in any way to projects containing study data. Participant data that is not self-report, such as blood cannabinoid levels, will be reported to us with excel sheets that only contain data points coded by participant ID and that are stored on our lab's password protected, CUB-based server (administered by OIT). This data is then transferred automatically (ETL) to REDCap through automated application program interface (API) calls between our server and REDCap. The API tokens are only assigned to the PI user account, and only shared to limited team members managing data for the purpose of imputing into the ETL scripts. The code itself is backed up to the lab's server and also in a secure Github repository and on a local, password protected PC in the lab. Therefore, any form of data or participant info we collect is only in REDCap or on our server, and portable devices will not be used for the storage of any data or participant information. At the end of the study, all links between participant name and coding number will be destroyed, at which point the data will be considered de-identified. Additionally, all other records will continue to be kept in a secure location for as long a period as dictated by IRB, Institutional, and sponsor regulations and requirements.

A study participant database for future research activities will be developed as a separate REDCap project. Individuals will be queried during the screening process as to whether they would like to be contacted for future research studies. If they indicate they would like to be contacted in the future, their contact information will be collected and stored in the separate project. A record of what research studies the individual has already been contacted about will also be stored to prevent redundant contacts. The individual will be allowed to be removed from the contact list at any time; if a participant requests not to be contacted again, we will delete their information from the REDCap project.

XIV. PROVISIONS TO PROTECT THE PRIVACY INTERESTS OF PARTICIPANTS

The privacy of study participants is of utmost importance to the study team. Participant screening, the informed consent process, and all subsequent study assessments, including blood draws, will be conducted either within private rooms in the CUChange Laboratory dedicated to participant study visits, or at the CTRC. Study therapists will all gain certification through the American Psychological Association's Continuing Education module on Telepsychology Best Practices for privacy and confidentiality when conducting telehealth therapy sessions (https://www.apa.org/career-development/telepsychology) as well as all guidelines set for by the State of Colorado for the governing of clinical psychotherapy in our state.

XV. WITHDRAWAL OF PARTICIPANTS

Situations in which the entire study may be terminated early include the following: If the Principal Investigator or other governing official discovers serious concerns about participants' safety; inadequate performance or rate of enrollment; because study objectives have been obtained according to pre-established statistical guidelines; or in the unlikely event that the Principal Investigator retires and no other additional investigators are able to succeed her role within the research project.

The circumstances under which a participant would be withdrawn without their consent (stopping criteria) include:

1. Participant not compliant with study procedures
2. Behavior by participant that is verbally or physically abusive towards research staff
3. AST or ALT levels elevated above two times the upper limit of the normal range when assessed at the Baseline or Week 4 Visit
4. Positive pregnancy test (females)
5. Breath alcohol level above 0.000 at any visit.
6. Positive urine toxicology screen for any substance of abuse besides alcohol, nicotine, or cannabis (e.g., cocaine, non-prescription use of opiates, methamphetamine, MDMA, benzodiazepines, or barbiturates).
7. High risk for suicide as classified by the C-SSRS or a response of "I would like to kill myself"
8. Adverse event or other safety concern that in the opinion of the PI or Study Physician would be in the best interest of the participant to discontinue study treatment

Those who experience early withdrawal will receive prorated payment based on the number of visits that they completed.

XVI. RISKS TO PARTICIPANTS

Participants may face several potential risks:

1. Unwanted side effects (e.g., nausea, diarrhea, somnolence, liver damage) may occur with use of CBD. Additionally, cannabis use may also increase the risk of becoming unstable/off-balance and thus, may increase the risk of falling. Although Epidiolex, which contains CBD, has been approved by the FDA for a rare seizure disorder in children, CBD is metabolized differently with expression of one metabolite that is much higher in humans than in animals. Animal safety studies have not been conducted with this metabolite and therefore the safety of this metabolite is unknown at this time. Based on studies in animals, CBD has been shown to cause male reproductive organ changes that can result in reduced male fertility. Also, CBD in animals have been associated with adverse effects to the fetus and fetal development.
2. There is a small risk of swelling, infection, and fainting associated with a blood draw.
3. Participants could face loss of confidentiality if the data they provide during the course of their study participation is linked to their name or other identifying information. Another example of a privacy breach would be if staff used an inappropriate means of communication with the participant (e.g., calling the participant instead of emailing them). Confidentiality may not be maintained if non-research staff obtained links to participant data and contact information or if research staff inappropriately disclosed participant records. Risks from breach of confidentiality or invasion of privacy have social and economic risks. Economic risks include alterations in relationships with others that are to the disadvantage of the participant, and may involve embarrassment, loss of respect of others, labeling with negative consequences, or diminishing the participant's opportunities and status in relation to others. These risks include payment by participants for procedures, loss of wages or income, and/or damage to employability or insurability. Participants will be asked and tested for illegal activities that they may have been involved in (i.e., illicit drug use). Participants will also be warned that there are some things that they might tell us that we CANNOT promise to keep confidential. Participants will be informed that we are required to report information like child abuse or neglect, crimes that they tell us they or others plan to commit, or harm planned against themselves or others.
4. Participants in the full spectrum CBD group could fail a drug test due to the small amount of THC present in the medication. A failed drug test could have economic consequences to the participant if drug testing is required for employment.
5. This study includes psychological risks while completing surveys, including fatigue and emotional discomfort while answering sensitive questions about medical history, substance use behaviors, anxiety, depression, and suicidality.

XVII. MANAGEMENT OF RISKS

1. CBD is available over the counter in most states in the U.S. and does not produce intoxicating effects. The most common adverse reactions associated with CBD are somnolence/sedation, nausea, and diarrhea. CBD may also be associated with hepatotoxicity, although this and other adverse events (except diarrhea) are primarily limited to patients with pediatric epilepsy on extremely high doses (1000 mg or more) of CBD[76]. To reduce these risks, we will follow the recommendations for drug-drug interactions in the Epidiolex label. Consistent with our other FDA-approved INDs, we will exclude individuals who are currently using anti-epileptic medications (e.g., clobazam, sodium valproate) or

medications known to have major interactions with Epidiolex (buprenorphine, leflunomide, levomethadyl acetate, lomitapide, mipomersen, pexidartinib, propoxyphene, sodium oxybate, and/or teriflunomide). In addition, all participants will be medically screened including a blood test for liver function, such that participants' liver function tests (alanine aminotransferase [ALT] and aspartate aminotransferase [AST]) must show levels no greater than 2x the upper normal limits at baseline to be included. While there are very few human studies of oral administration of plant-derived CBD, a recent dose-ranging study found that doses up to 240mg produced minimal side effects and were well tolerated[77]. It is also important to note that, unlike synthetic CBD, plant-derived full spectrum CBD contains a range of cannabinoids and other bioactive ingredients that may impact bioavailability, efficacy, and adverse effects. This is critical as we seek to compare full spectrum CBD (CBD+THC) to broad spectrum CBD (CBD only) and match the dose of CBD given in each condition. Given this information, we chose to test a CBD dose of 400mg daily (taken in two 200mg doses, one in the morning and one in the evening), which is low enough not to produce side effects but should be high enough to see potential effects on our outcomes. Finally, we will provide participants with safety information that will contain warnings regarding driving or operation of machinery until they know how their assigned medication affects them.

2. The risks of bruising and infection are minimized by having trained personnel perform the procedures using sterile techniques. We will also provide snacks and water, and participants will be supervised in a seated and protected position during each blood draw and will remain seated after each until all symptoms (e.g., dizziness) are resolved to reduce the risk of falling.
3. Fully informed consent will be sought to ensure that participants are aware of any possible risks regarding confidentiality and privacy. Regarding confidentiality, we intend to mitigate risks as much as possible by collecting the minimum amount of identifying information from participants necessary to conduct our study. Participants' information will be coded with a number, and the document linking their number with their contact information will be stored on a password protected server that is only accessible by members of the research staff. All study computers are password protected and housed in the PI's lab space, which is kept locked unless researchers are currently using the space. All identifying information (e.g., consent forms, contact information) is kept separate and secure from the data files. Any identifying information and biological samples will be destroyed after all analyses are complete. After this, there will be no way to connect participant's names with participant data, at which point they will be considered de-identified. Lastly, to avoid a breach of privacy via inappropriate means of communication, participants will have the opportunity to decide what form of communication (e.g., phone, email, text) they would like to be contacted at throughout the study. If they choose to be contacted by phone, we will ask if a researcher can leave a voicemail on the number provided.
4. Participants will be made aware of the risk of failing a drug test during the informed consent process. They will be made aware that one of the medications contains small amounts of THC, which could show up in a drug test. They will also be made aware that they will not know which medication they are assigned to during the study.
5. Participants will be allowed breaks while completing surveys if they experience fatigue. Participants experiencing emotional discomfort while completing surveys about sensitive topics will be reminded that participation in the research is voluntary, including providing answers to individual questions.

XVIII. POTENTIAL BENEFITS

Participants may benefit from learning more about the health effects of CBD.

XIX. PROVISIONS TO MONITOR THE DATA FOR THE SAFETY OF PARTICIPANTS

The study coordinators listed as Key Personnel in this document will monitor and report to the PI on adherence to the protocol. They will assess adherence via periodic observation of the visits and visual inspection of the completeness of data collection. In addition, the PI will be in daily contact with the research assistants conducting study visits and the study therapists administering the psychotherapy sessions and will be informed immediately of any adverse event. Adverse events will be queried at each study visit by asking participants if they have experienced any new and unwanted changes in their physical or mental health, as well as if they have visited an urgent care or emergency room, been hospitalized, or undergone any unplanned medical procedures or surgeries since their previous visit. Information to be collected about adverse events will include event description, time of onset (start date), and time of resolution/stabilization of the event (end date), which will recorded in an Adverse Event Log by a research assistant, research coordinator, or study therapist. In addition, the Study Physician will review all adverse events to determine the relationship of the adverse event to the study intervention, the severity of the adverse event, and the expectedness of the adverse event. The Study Physician will also determine whether it is safe for the participant continue study participation. The Study Physician will be blinded to condition assignment, and the blind will only be broken in emergencies when knowledge of the participant's group is necessary for participant safety. Consistent with IRB policy, a study coordinator will use the Event Reporting (New Information) eForm in eRA to report harms experienced by participants that are unexpected and probably related to the research procedures, protocol deviations, and other qualifying events within five business days of the PI becoming aware of the issue. All other harms experienced by participants will be reported annually to the IRB at the continuing review. In addition, the Study Physician will regularly review aggregate adverse event data and other safety data such as liver function tests to monitor the ongoing safety of the trial. Annual reports of safety information will be prepared following FDA format by the Study Physician with assistance from the study team for submission to the FDA and the IRB. A detailed description of adverse event and serious adverse event determination and reporting is provided as a separate document (Lab Management for Determining and Reporting Adverse Events).

Another potential risk that the study team will be prepared to manage is participant endorsement of thoughts of suicide on the C-SSRS or the MINI. The C-SSRS is a measure of suicidality, and the MINI includes suicidality assessment. To ensure the safety of our participants, a research assistant will check the participant's response to the C-SSRS before the participant leaves the building. If the participant is categorized as high risk based on their responses to the C-SSRS, or if suicidality is indicated during the MINI, the research assistant will immediately notify PI Bidwell (a licensed clinical psychologist). Dr. Bidwell will immediately assess the participant for imminent suicide risk and triage the participant depending on their acuity, including options of placing them on clinical hold for imminent suicide risk and/or providing them with a list of psychological services referral contacts for less acute levels of risk. A research coordinator will immediately follow up with the PI to ensure that all of the necessary measures have been taken to protect the safety of the participant. During this meeting, the PI and research coordinator, will determine whether it is safe for the participant to continue involvement in the study. If it is determined that the participant is at imminent risk and should not continue participating in the study, the study coordinator will contact the participant and explain that for their safety, the research team does not feel that it is safe for them to continue participating in the study. The study coordinator will also answer any questions that the participant may have about this decision.

In addition, NIDA has requested that a Data Safety Monitoring Board (DSMB) be formed for this project to assure participant safety and data quality. The DSMB includes three experts in CUD, CBD pharmacology, and clinical trials, none of whom have financial conflicts with the results of the trial. A detailed Data Safety Monitoring plan is included as a separate document. All DSMB reports and any required responses to the DSMB will be submitted to the IRB.

XX. MEDICAL CARE AND COMPENSATION FOR INJURY

Participants will be informed to contact Dr. Bidwell immediately by phone (303-492-9549) should they feel that they have been harmed while participating in this study. They will be told that the cost for any treatment will be billed to them or their medical or hospital insurance. Information regarding compensation for injury is included in the informed consent document.

XXI. COST TO PARTICIPANTS

There are no anticipated costs. Parking is free at the CINC, and participants will be provided with study products.

XXII. DRUG ADMINISTRATION

The PI will adhere to the requirements set forth in 21 CFR Part 312.

XXIII. INVESTIGATIONAL DEVICES

N/A.

XXIV. WORKING WITH OTHER INSTITUTIONS

All data collection procedures will occur at CU Boulder by CU Boulder personnel. The Study Physician at CU Anschutz will review participant medication use, medical history, and blood tests collected by CU Boulder personnel to confirm eligibility prior to initiation of study medication use via REDCap. The Study Physician will also have access to the participant identifiers in REDCap but will be blinded to condition assignment. The blind will only be broken in emergencies when knowledge of the participant’s group is necessary for participant safety. The Study Physician will also review aggregate adverse event and liver function test data annually in preparation to submit the annual report to the FDA.

XXV. SHARING OF RESULTS WITH PARTICIPANTS

The results of clinical blood tests (CMP and CBC) will be made available to participants upon request. Any clinically relevant research results (e.g., abnormal CMP or CBC) will be disclosed to participants.

XXVI. REFERENCES

1. Bidwell LC, YorkWilliams SL, Mueller R, Bryan AD, Hutchison KE. Exploring cannabis concentrates on the legal market: User profiles, product strength, and health-related outcomes. *Addictive Behaviors Reports*. 2018;8(March):102-106. doi:10.1016/j.abrep.2018.08.004
2. Raber JC, Elzinga S, Kaplan C. Understanding dabs: contamination concerns of cannabis concentrates and cannabinoid transfer during the act of dabbing. *J Toxicol Sci*. 2015;40(6):797-803. doi:10.2131/jts.40.797
3. Bidwell LC, Ellingson JM, Karoly HC, et al. Association of naturalistic administration of cannabis flower and concentrates with intoxication and impairment. *JAMA Psychiatry*. 2020;77(8):787-796. doi:10.1001/jamapsychiatry.2020.0927
4. Batalla A, Janssen H, Gangadin SS, Bossong MG. The Potential of Cannabidiol as a Treatment for Psychosis and Addiction: Who Benefits Most? A Systematic Review. *J Clin Med*. 2019;8(7). doi:10.3390/jcm8071058
5. Brezing CA, Levin FR. The Current State of Pharmacological Treatments for Cannabis Use Disorder and Withdrawal. *Neuropsychopharmacology 2018 43:1*. 2017;43(1):173-194. doi:10.1038/npp.2017.212
6. Drennan ML, Karoly HC, Bryan AD, Hutchison KE, Bidwell LC. Acute objective and subjective intoxication effects of legal-market high potency THC-dominant versus CBD-dominant cannabis concentrates. *Scientific Reports 2021 11:1*. 2021;11(1):1-10. doi:10.1038/s41598-021-01128-2
7. Volkow ND, Baler RD, Compton WM, Weiss SRB. Adverse health effects of marijuana use. *N Engl J Med*. 2014;370(23):2219-2227. doi:10.1056/NEJMra1402309
8. Al-Zouabi I, Stogner JM, Miller BL, Lane ES. Butane hash oil and dabbing: insights into use, amateur production techniques, and potential harm mitigation. *Subst Abuse Rehabil*. 2018;9:91-101. doi:10.2147/SAR.S135252
9. Di Forti M, Sallis H, Allegri F, et al. Daily Use, Especially of High-Potency Cannabis, Drives the Earlier Onset of Psychosis in Cannabis Users. *Schizophr Bull*. 2014;40(6):1509-1517. doi:10.1093/schbul/sbt181
10. Pierre JM. Risk of Increasingly Potent Cannabis. *Curr Psychiatr*. 2017;16(2):15-20.
11. Arterberry BJ, Treloar Padovano H, Foster KT, Zucker RA, Hicks BM. Higher average potency across the United States is associated with progression to first cannabis use disorder symptom. *Drug Alcohol Depend*. 2019;195:186-192. doi:10.1016/j.drugalcdep.2018.11.012
12. Di Forti M, Marconi A, Carra E, et al. Proportion of patients in south London with first-episode psychosis attributable to use of high potency cannabis: a case-control study. *Lancet Psychiatry*. 2015;2(3):233-238. doi:10.1016/S2215-0366(14)00117-5
13. Freeman TP, Winstock AR. Examining the profile of high-potency cannabis and its association with severity of cannabis dependence. *Psychol Med*. 2015;45(15):3181-3189. doi:10.1017/S0033291715001178
14. Loflin M, Earleywine M. A new method of cannabis ingestion: The dangers of dabs? *Addictive Behaviors*. 2014;39(10):1430-1433. doi:10.1016/J.ADDBEH.2014.05.013
15. Meier MH. Associations between butane hash oil use and cannabis-related problems. *Drug Alcohol Depend*. 2017;179:25-31. doi:10.1016/J.DRUGALCDEP.2017.06.015
16. Pierre JM, Gandal M, Son M. Cannabis-induced psychosis associated with high potency “wax dabs.” *Schizophr Res*. 2016;172(1-3):211-212. doi:10.1016/J.SCHRES.2016.01.056
17. Barrington-Trimis JL, Cho J, Ewusi-Boisvert E, et al. Risk of Persistence and Progression of Use of 5 Cannabis Products After Experimentation Among Adolescents. *JAMA Netw Open*. 2020;3(1):e1919792. doi:10.1001/jamanetworkopen.2019.19792

18. D'Souza DC, Cortes-Briones JA, Ranganathan M, et al. Rapid Changes in Cannabinoid 1 Receptor Availability in Cannabis-Dependent Male Subjects After Abstinence From Cannabis. *Biol Psychiatry Cogn Neurosci Neuroimaging*. 2016;1(1):60-67. doi:10.1016/j.bpsc.2015.09.008
19. Hirvonen J, Goodwin RS, Li CT, et al. Reversible and regionally selective downregulation of brain cannabinoid CB1 receptors in chronic daily cannabis smokers. *Mol Psychiatry*. 2012;17(6):642-649. doi:10.1038/mp.2011.82
20. Navarrete F, García-Gutiérrez MS, Gasparyan A, Austrich-Olivares A, Manzanares J. Role of Cannabidiol in the Therapeutic Intervention for Substance Use Disorders. *Front Pharmacol*. 2021;12. doi:10.3389/fphar.2021.626010
21. Cunha JM, Carlini EA, Pereira AE, et al. Chronic Administration of Cannabidiol to Healthy Volunteers and Epileptic Patients. *Pharmacology*. 1980;21(3):175-185. doi:10.1159/000137430
22. Bhattacharyya S, Morrison PD, Fusar-Poli P, et al. Opposite Effects of Δ-9-Tetrahydrocannabinol and Cannabidiol on Human Brain Function and Psychopathology. *Neuropsychopharmacology*. 2010;35(3):764-774. doi:10.1038/npp.2009.184
23. Babalonis S, Haney M, Malcolm RJ, et al. Oral cannabidiol does not produce a signal for abuse liability in frequent marijuana smokers. *Drug Alcohol Depend*. 2017;172:9-13. doi:10.1016/j.drugalcdep.2016.11.030
24. Herkenham M, Lynn AB, Johnson MR, Melvin LS, De Costa BR, Rice KC. *Characterization and Localization of Cannabinoid Receptors in Rat Brain: A Quantitative in Vitro Autoradiographic Study*. Vol 7.; 1991.
25. Sloan ME, Gowin JL, Ramchandani VA, Hurd YL, Le Foll B. The endocannabinoid system as a target for addiction treatment: Trials and tribulations. *Neuropharmacology*. 2017;124:73-83. doi:10.1016/j.neuropharm.2017.05.031
26. Campos AC, Moreira FA, Gomes F V., Del Bel EA, Guimaraes FS. Multiple mechanisms involved in the large-spectrum therapeutic potential of cannabidiol in psychiatric disorders. *Philosophical Transactions of the Royal Society B: Biological Sciences*. 2012;367(1607):3364-3378. doi:10.1098/rstb.2011.0389
27. Devinsky O, Cilio MR, Cross H, et al. Cannabidiol: Pharmacology and potential therapeutic role in epilepsy and other neuropsychiatric disorders. *Epilepsia*. 2014;55(6):791-802. doi:10.1111/epi.12631
28. Bergamaschi MM, Queiroz RHC, Chagas MHN, et al. Cannabidiol Reduces the Anxiety Induced by Simulated Public Speaking in Treatment-Naïve Social Phobia Patients. *Neuropsychopharmacology*. 2011;36(6):1219-1226. doi:10.1038/npp.2011.6
29. Ren Y, Whittard J, Higuera-Matas A, Morris C V., Hurd YL. Cannabidiol, a nonpsychotropic component of cannabis, inhibits cue-induced heroin seeking and normalizes discrete mesolimbic neuronal disturbances. *Journal of Neuroscience*. 2009;29(47):14764-14769. doi:10.1523/JNEUROSCI.4291-09.2009
30. Hurd YL, Spriggs S, Alishayev J, et al. Cannabidiol for the reduction of cue-induced craving and anxiety in drug-abstinent individuals with heroin use disorder: A double-blind randomized placebo-controlled trial. *American Journal of Psychiatry*. Published online 2019. doi:10.1176/appi.ajp.2019.18101191
31. Haney M, Cooper ZD, Bedi G, Vosburg SK, Comer SD, Foltin RW. Nabilone decreases marijuana withdrawal and a laboratory measure of marijuana relapse. *Neuropsychopharmacology*. 2013;38(8):1557-1565. doi:10.1038/npp.2013.54
32. Trigo JM, Lagzdins D, Rehm J, et al. Effects of fixed or self-titrated dosages of Sativex on cannabis withdrawal and cravings. *Drug Alcohol Depend*. 2016;161:298-306. doi:10.1016/j.drugalcdep.2016.02.020

33. Allsop DJ, Copeland J, Lintzeris N, et al. Nabiximols as an Agonist Replacement Therapy During Cannabis Withdrawal. *JAMA Psychiatry*. 2014;71(3):281. doi:10.1001/jamapsychiatry.2013.3947
34. Lintzeris N, Bhardwaj A, Mills L, et al. Nabiximols for the Treatment of Cannabis Dependence: A Randomized Clinical Trial. *JAMA Intern Med*. 2019;179(9):1242-1253. doi:10.1001/jamainternmed.2019.1993
35. Freeman TP, Hindocha C, Baio G, et al. Cannabidiol for the treatment of cannabis use disorder: a phase 2a, double-blind, placebo-controlled, randomised, adaptive Bayesian trial. *Lancet Psychiatry*. 2020;7(10):865-874. doi:10.1016/S2215-0366(20)30290-X
36. Haney M, Malcolm RJ, Babalonis S, et al. Oral Cannabidiol does not Alter the Subjective, Reinforcing or Cardiovascular Effects of Smoked Cannabis. *Neuropsychopharmacology*. 2016;41(8):1974-1982. doi:10.1038/npp.2015.367
37. Solowij N, Broyd SJ, Beale C, et al. Therapeutic Effects of Prolonged Cannabidiol Treatment on Psychological Symptoms and Cognitive Function in Regular Cannabis Users: A Pragmatic Open-Label Clinical Trial. *Cannabis Cannabinoid Res*. 2018;3(1):21-34. doi:10.1089/can.2017.0043
38. Trigo JM, Soliman A, Quilty LC, et al. Nabiximols combined with motivational enhancement/cognitive behavioral therapy for the treatment of cannabis dependence: A pilot randomized clinical trial. *PLoS One*. 2018;13(1):e0190768. doi:10.1371/JOURNAL.PONE.0190768
39. Gallily R, Yekhtin Z, Ondřej Hanuš L. Overcoming the Bell-Shaped Dose-Response of Cannabidiol by Using Cannabis Extract Enriched in Cannabidiol. *Pharmacol Pharm*. 2015;6:75-85. doi:10.4236/pp.2015.62010
40. Steeger CM, Hitchcock LN, Bryan AD, Hutchison KE, Hill KG, Bidwell LC. Associations between self-reported cannabis use frequency, potency, and cannabis/health metrics. *International Journal of Drug Policy*. 2021;97:103278. doi:10.1016/j.drugpo.2021.103278
41. Bidwell LC, Mueller R, YorkWilliams S, Hagerty SL, Bryan AD, Hutchison KE. A novel observational method for assessing acute responses to cannabis: Preliminary validation using legal market strains. *Cannabis Cannabinoid Res*. Published online 2018.
42. Lovibond SH, Lovibond PF. *Manual for the Depression Anxiety Stress Scales*.; 1996.
43. Posner K, Brown GK, Stanley B, et al. The Columbia–Suicide Severity Rating Scale: Initial Validity and Internal Consistency Findings From Three Multisite Studies With Adolescents and Adults. *Am J Psychiatry*. 2011;(168):1266-1277. www.cssrs.columbia.edu
44. Stephens RS, Roffman RA, Curtin L. Comparison of extended versus brief treatments for marijuana use. *J Consult Clin Psychol*. 2000;68(5):898-908. doi:10.1037/0022-006X.68.5.898
45. Adamson SJ, Kay-Lambkin FJ, Baker AL, et al. An improved brief measure of cannabis misuse: the Cannabis Use Disorders Identification Test-Revised (CUDIT-R). *Drug Alcohol Depend*. 2010;110(1-2):137-143. doi:10.1016/j.drugalcdep.2010.02.017
46. Budney AJ, Moore BA, Vandrey RG, Hughes JR. The time course and significance of cannabis withdrawal. *J Abnorm Psychol*. 2003;112(3):393-402. doi:10.1037/0021-843x.112.3.393
47. Davis CN, Gex KS, Squeglia LM, et al. Development and Initial Validation of a Momentary Cannabis Craving Scale Within a Homogeneous Sample of U.S. Emerging Adults. *Assessment*. Published online March 21, 2024. doi:10.1177/10731911241237055
48. Schafer J, Brown SA. Marijuana and Cocaine Effect Expectancies and Drug Use Patterns. *J Consult Clin Psychol*. 1991;59(4):558-565. doi:10.1037/0022-006X.59.4.558
49. Weiss JH, Tervo-Clemmens B, Potter KW, Evins AE, Gilman JM. The Cannabis Effects Expectancy Questionnaire–Medical (CEEQ-M): Preliminary psychometric properties and longitudinal validation within a clinical trial. *Psychol Assess*. Published online June 8, 2023. doi:10.1037/pas0001244

50. Cuttler C, Spradlin A. Measuring cannabis consumption: Psychometric properties of the Daily Sessions, Frequency, Age of Onset, and Quantity of Cannabis Use Inventory (DFAQ-CU). *PLoS One*. 2017;12(5). doi:10.1371/journal.pone.0178194
51. Babor TF, Higgins-Biddle JC, Saunders JB, Monteiro MG. The Alcohol Use Disorders Identification Test Guidelines for Use in Primary Care. *World Health Organization*. Published online 2001:pp1-40. doi:10.1177/0269881110393051
52. Sobell LC, Sobell MB. Timeline Follow-Back. *Measuring Alcohol Consumption*. Published online 1992:41-72. doi:10.1007/978-1-4612-0357-5_3
53. Sobell MB, Sobell LC, VanderSpek R. Relationship between clinical judgment, self-report, and breath-analysis measures of intoxication in alcoholics. *J Consult Clin Psychol*. 1979;47(1):204-206. doi:10.1037/0022-006x.47.1.204
54. Martin-Willett R, McCormick Z, Newman W, Larsen LD, Ortiz Torres MA, Bidwell LC. The transformation of a gold standard in-person substance use assessment to a web-based, REDCap integrated data capture tool. *J Biomed Inform*. 2019;94:103186. doi:10.1016/J.JBI.2019.103186
55. Klawitter J, Sempio C, Morlein S, et al. An Atmospheric Pressure Chemical Ionization MS/MS Assay Using Online Extraction for the Analysis of 11 Cannabinoids and Metabolites in Human Plasma and Urine. *Ther Drug Monit*. 2017;39(5):556-564. doi:10.1097/FTD.0000000000000427
56. Saunders JB, Aasland OG, Babor TF, De la Fuente JR, Grant M. Development of the Alcohol Use Disorders Identification Test (AUDIT): WHO Collaborative Project on Early Detection of Persons with Harmful Alcohol Consumption-II. *Addiction*. 1993;88(6):791-804. doi:10.1111/j.1360-0443.1993.tb02093.x
57. Beck AT. *Beck Anxiety Inventory®*. 2nd ed. Pearson (Psychological Corporation); 1993. Accessed March 19, 2018. https://www.pearsonclinical.com/psychology/products/100000251/beck-anxiety-inventory-bai.html
58. Beck AT, Steer RA, Brown GK. *Beck Depression Inventory®-II*. Pearson (Psychological Corporation); 1996. Accessed March 19, 2018. https://www.pearsonclinical.com/psychology/products/100000159/beck-depression-inventoryii-bdi-ii.html
59. Turner-Bowker D, Hogue SJ. Short Form 12 Health Survey (SF-12). In: *Encyclopedia of Quality of Life and Well-Being Research*. Springer Netherlands; 2014:5954-5957. doi:10.1007/978-94-007-0753-5_2698
60. Stephens RS, Roffman RA, Curtin L. Comparison of extended versus brief treatments for marijuana use. *J Consult Clin Psychol*. 2000;68(5):898-908. https://www.ncbi.nlm.nih.gov/pubmed/11068976
61. Sobell L, Sobell M. Timeline follow-back: A technique for assessing self-reported alcohol consumption. In: *Measuring Alcohol Consumption*. ; 1992:41-72. doi:10.1007/978-1-4612-0357-5_3
62. Budney A, Borodovsky J, Struble CA, et al. Estimating THC consumption from smoked and vaped cannabis products in an online survey of adults who use cannabis. *Cannabis Cannabinoid Res*. Published online 2022.
63. MEMS® adherence hardware solution - AARDEX Group. Accessed December 4, 2022. https://www.aardexgroup.com/solutions/mems-adherence-hardware/
64. FDA, CDER. Enrichment Strategies for Clinical Trials to Support Determination of Effectiveness of Human Drugs and Biological Products Guidance for Industry. Published online March 2019. Accessed December 4, 2022. https://www.fda.gov/Drugs/GuidanceComplianceRegulatoryInformation/Guidances/default.htm and/orhttps://www.fda.gov/BiologicsBloodVaccines/GuidanceComplianceRegulatoryInformation/Guidances/default.htm

65. Shrout PE, Bolger N. Mediation in experimental and nonexperimental studies: New procedures and recommendations. *Psychol Methods*. 2002;7(4):422-445. doi:10.1037//1082-989X.7.4.422
66. Schafer JL, Graham JW. Missing data: our view of the state of the art. *Psychol Methods*. 2002;7(2):147-177. Accessed June 11, 2018. http://www.ncbi.nlm.nih.gov/pubmed/12090408
67. Benjamini Y, Hochberg Y. *Controlling the False Discovery Rate: A Practical and Powerful Approach to Multiple Testing*. Vol 57.; 1995.
68. Schmiege SJ, Broaddus MR, Levin M, Bryan AD. Randomized trial of group interventions to reduce HIV/STD risk and change theoretical mediators among detained adolescents. *J Consult Clin Psychol*. 2009;77(1):38-50. doi:10.1037/a0014513
69. Bryan A, Schmiege SJ, Broaddus MR. Mediational Analysis in HIV/AIDS Research: Estimating Multivariate Path Analytic Models in a Structural Equation Modeling Framework. *AIDS Behav*. 2007;11(3):365-383. doi:10.1007/s10461-006-9150-2
70. MacKinnon D, Lockwood C, Williams J. Confidence Limits for the Indirect Effect: Distribution of the Product and Resampling Methods. *Multivariate Behav Res*. 2004;39(1):99-128. doi:10.1207/s15327906mbr3901
71. Ruiz CM, Torrens A, Castillo E, et al. Pharmacokinetic, behavioral, and brain activity effects of ?9-tetrahydrocannabinol in adolescent male and female rats. *Neuropsychopharmacology*. 2021;46(5):959-969. doi:10.1038/s41386-020-00839-w
72. Ehlers CL, Gizer IR, Vieten C, et al. Cannabis dependence in the San Francisco Family Study: Age of onset of use, DSM-IV symptoms, withdrawal, and heritability. *Addictive Behaviors*. 2010;35(2):102-110. doi:10.1016/j.addbeh.2009.09.009
73. Schlienz NJ, Budney AJ, Lee DC, Vandrey R. Cannabis Withdrawal: a Review of Neurobiological Mechanisms and Sex Differences. *Curr Addict Rep*. 2017;4(2):75-81. doi:10.1007/s40429-017-0143-1
74. Sholler DJ, Strickland JC, Spindle TR, Weerts EM, Vandrey R. Sex differences in the acute effects of oral and vaporized cannabis among healthy adults. *Addiction Biology*. 2020;(April 2020):1-12. doi:10.1111/adb.12968
75. Drennan ML, Karoly HC, Bryan AD, Hutchison KE, Bidwell LC. Acute objective and subjective intoxication effects of legal-market high potency THC-dominant versus CBD-dominant cannabis concentrates. *Scientific Reports 2021 11:1*. 2021;11(1):1-10. doi:10.1038/s41598-021-01128-2
76. Chesney E, Oliver D, Green A, et al. Adverse effects of cannabidiol: a systematic review and meta-analysis of randomized clinical trials. *Neuropsychopharmacology*. 2020;45(11):1799-1806. doi:10.1038/s41386-020-0667-2
77. Amor S, Puentes F, Baker D, Van Der Valk P. Inflammation in neurodegenerative diseases. *Immunology*. 2010;129(2):154-169. doi:10.1111/j.1365-2567.2009.03225.x
